# Differential immune responses in pregnant patients recovered from COVID-19

**DOI:** 10.1038/s41392-021-00703-3

**Published:** 2021-07-29

**Authors:** Ge Chen, Yiming Zhang, Yaoyao Zhang, Jihui Ai, Bin Yang, Mengge Cui, Qiuyue Liao, Hanxiao Chen, Hualin Bai, Dashing Shang, Jing Chen, ChaoYang Sun, Haiyi Liu, Fengyuan Liu, Bin Mao, Guoqiang Sun, Lu Chen, Jing-wen Lin, Kezhen Li

**Affiliations:** 1 Tongji Hospital, Tongji Medical College, Huazhong University of Science and Technology, Wuhan, Hubei China; 2 Key Laboratory of Birth Defects and Related Diseases of Women and Children of MOE, State Key Laboratory of Biotherapy, West China Second University Hospital, Sichuan University and Collaborative Innovation Center for Biotherapy, Chengdu, Sichuan China; 3 Biological Sciences Greenhouse, College of Art & Sciences, The Ohio State University, Columbus, OH USA; 4 Department of Obstetrics, Hubei Maternal and Child Health Hospital, Wuhan, China

**Keywords:** Infectious diseases, Innate immune cells

## Abstract

Pregnant women are generally more susceptible to viral infection. Although the impact of SARS-CoV-2 in pregnancy remains to be determined, evidence indicates that the risk factors for severe COVID-19 are similar in pregnancy to the general population. Here we systemically analyzed the clinical characteristics of pregnant and non-pregnant female COVID-19 patients who were hospitalized during the same period and found that pregnant patients developed marked lymphopenia and higher inflammation evident by higher C-reactive protein and IL-6. To elucidate the pathways that might contribute to immunopathology or protective immunity against COVID-19 during pregnancy, we applied single-cell mRNA sequencing to profile peripheral blood mononuclear cells from four pregnant and six non-pregnant female patients after recovery along with four pregnant and three non-pregnant healthy donors. We found normal clonal expansion of T cells in the pregnant patients, heightened activation and chemotaxis in NK, NKT, and MAIT cells, and differential interferon responses in the monocyte compartment. Our data present a unique feature in both innate and adaptive immune responses in pregnant patients recovered from COVID-19.

## Introduction

Coronavirus disease 2019 (COVID-19) has become a global pandemic. As of 16 February 2021, there were 103,362,039 confirmed cases and 2,244,713 deaths, according to World Health Organization.^1^ COVID-19 patients present diverse clinical manifestations, ranging from asymptomatic to critical that requires mechanical ventilation.^2,3^ Multi-system injuries and death observed in patients are thought to be associated with immune dysfunctions induced by severe acute respiratory syndrome coronavirus 2 (SARS-CoV-2),^4,5^ and appropriate immune response might play an important role in convalescence. Despite the progress, research into immune responses against the infection remains important in delineating the mechanisms underlying immunopathology leading to severe COVID-19.

Pregnant women are generally more susceptible to viral infection.^6,7^ A notable increase of deaths was documented in pregnant patients during 2003 SARS pandemic that was caused by SARS-CoV-1.^8^ Currently, there are many questions regarding the vulnerability of pregnant women to SARS-CoV2 infection.^9^ It is reported that pregnant women with COVID-19 were more likely to be transferred to the Intensive Care Unit and require mechanical ventilation in the US.^10^ A systematic review and meta-analysis of mainly small case series reported that a high proportion of women with confirmed COVID-19 infection had preterm birth and cesarean delivery.^11^ However, the current evidence suggests that pregnant women are neither more susceptible to SARS-CoV-2 infection nor tend to develop more severe symptoms than non-pregnant patients upon infection.^12–14^ Epidemiological data from China,^15^ UK,^16^ and France^17^ presented that only a small fraction of pregnant patients developed severe disease, and mortality from COVID-19 was rarely reported.

Pregnancy comprises a unique immunological status, to protect the fetus from maternal rejection, allowing adequate fetal development while maintaining the resistance to exogenous pathogens. Features in immune states during pregnancy are often characterized by alterations in the cellular composition and the functions of immune cells, including suppression of T cell-mediated immunity and humoral responses, particularly during the third trimester.^18–23^ On the other hand, innate responses, in particular progressively activated monocytes, were noted in the circulation during pregnancy.^22,24^ It was reported that influenza A virus infection during pregnancy exaggerated inflammatory response of monocytes and plasmacytoid dendritic cells (pDCs).^25^ T cells and natural killer (NK) cells also displayed enhanced functional responses and elevated interferon (IFN)-γ production upon H1N1 challenge.^26,27^ However, how pregnancy affects immune responses against SARS-CoV-2 infection is largely unknown.

The unbiased high-throughput single-cell RNA sequencing (scRNA-seq) technologies with high accuracy and specificity revealed several cell types play important roles in COVID-19. Many cell types have been implicated as important for COVID-19 pathogenesis or immune protection, such as monocytes,^28,29^ NK,^30^ mucosal-associated invariant T cell (MAIT),^31^ and T and B cells.^32–35^ B cell receptor (BCR) and T cell receptor (TCR) V(D)J gene rearrangements and clone expansion stimulated by SARS-CoV-2 infection were also observed across different severities.^36^ Meanwhile, pro-inflammatory genes have been found elevated in several studies,^35^ especially type I IFN response in multiple cell subsets.^37^ Here we implemented scRNA-seq and single-cell TCR V(D)J sequencing to characterize the immune status of pregnant COVID-19 patients in the recovered phase. We identified differential IFN and innate responses and stronger chemotaxis signature in multiple cell types from the recovered pregnant patients. These findings may help to extend our understanding of how the immune alteration in pregnancy affects immune responses against SARS-CoV-2 infection.

## Results

### Higher inflammatory responses in pregnant COVID-19 patients

Between March 18 and May 9 2020, 20 pregnant (PCov) and 159 non-pregnant (NCov) female COVID-19 patients were admitted to Tongji Hospital. The clinical symptoms and laboratory measurements at admission are listed in Table 1. The pregnant patients were generally more anemic than their non-pregnant counterparts with lower hemoglobin levels and lower hematocrit. While total white blood cells and neutrophils were higher in the pregnant patients, the number of lymphocytes was significantly lower (Table 1). The higher level of neutrophils was maintained for the first 3 weeks after symptom onset while the trend of reduced lymphocyte level was persistent throughout the infection (Supplementary Fig. 1). Lower urea and creatinine were observed in the pregnant patients, as well as shorter prothrombin time (Table 1 and Supplementary Fig. 1). Notably, interleukin (IL)-6, an inflammatory cytokine that was found associated with more adverse clinical outcomes for COVID-19,^38^ was 3.5 times higher in the pregnant patients. Correspondingly, high-sensitivity C-reactive protein (hs-CRP) was nearly seven times higher in the pregnant patients compared to the non-pregnant patients, and higher D-dimer was also observed with the pregnant groups. Lymphopenia and elevated hs-CRP and D-dimer indicate elevated inflammation and were reported to be associated with higher severity of COVID-19.^39,40^ However, in our cohort, the percentage of the pregnant patients who developed severe COVID-19 symptoms was no higher than the non-pregnant female patients (Supplementary Table 1), in consistent with previous reports.^16,41^

**Table 1:** Clinical characteristics of COVID-19 patients and laboratory tests

Characteristic	Pregnant COVID-19	Non-pregnant COVID-19	*p*^a^
	*n* = 20	*n* = 159	
Median age, IQR (years)	30.5 (27.5-34.8)	33.0 (29.0-37.0)	
Comorbidity, no. (%) of patients			
Any	2 (10)	21 (13)	
Hypertension	0 (0)	5 (3)	
Diabetes	0 (0)	5 (3)	
Cardiovascular diseases	0 (0)	2 (1)	
Hepatitis or fatty liver	1 (5)	4 (3)	
Chronic bronchitis, bronchial asthma	1 (5)	3 (2)	
Malignancy	0 (0)	2 (1)	
Severe/critical cases, no. (%) of patients	1 (5)	23 (14)	
Viral shedding time (days)	15.0 (12.0–28.0)	33.0 (21.0–45.0)	0.057
Hospital staying(days)	16.5 (11.3–28.0)	14.0 (10.0–21.8)	0.218
Baseline on admission			
Temperature (°C)	36.80 (36.35–37.80)	36.60 (36.30–36.90)	0.215
Diastolic pressure (mm/Hg)	76.00 (70.75–83.50)	79.00 (71.00–87.00)	0.180
Systolic pressure (mm/Hg)	114.50 (107.25–126.75)	116.00 (106.00–125.00)	0.905
Respiratory rate (*n*/min)	20.00 (20.00–21.00)	20.00 (19.00–20.00)	**0.046**
Blood routine	*n* = 20	*n* = 159	
WBC (×10^9^/L)	7.85 (5.96–9.14)	5.27 (3.89–6.61)	**0.000**
Neutrophil (×10^9^/L)	5.61 (4.32–7.54)	3.08 (2.15–4.01)	**0.000**
Neutrophil (%)	0.75 (0.70–0.83)	0.58 (0.51–0.64)	**0.000**
Lymphocyte (×10^9^/L)	1.33 (0.94–1.60)	1.63 (1.08–2.01)	**0.042**
Lymphocyte (%)	0.19 (0.13–0.21)	0.31 (0.25–0.38)	**0.000**
Monocyte (×10^9^/L)	0.49 (0.34–0.62)	0.42 (0.32–0.56)	0.209
Monocyte (%)	0.06 (0.05–0.08)	0.08 (0.07–0.10)	0.081
Hematocrit (%)	33.25 (31.53–35.70)	36.80 (34.60–39.00)	**0.001**
Hemoglobin (g/L)	112.50 (101.50–125.75)	125.00 (119.00–133.00)	**0.005**
Platelet (×10^9^/L)	196.50 (141.50–246.50)	225.00 (177.00–268.000	0.129
hs-CRP (mg/L)	15.90 (2.58–36.35)	2.30 (0.50–13.00)	**0.004**
Serum biochemistry			
ALT (U/L)	12.00 (8.25–19.00)	13.00 (9.00–24.00)	0.346
AST (U/L)	17.00 (14.25–22.00)	18.00 (14.00–24.00)	0.750
TBIL (μmol/L)	6.15 (5.03–8.08)	6.40 (4.40–8.70)	0.700
DBIL (μmol/L)	3.15 (2.48–3.88)	2.70 (2.10–3.70)	0.089
IBIL (μmol/L)	2.85 (1.83–4.03)	3.60 (2.30–5.30)	0.051
Albumin (g/L)	34.80 (31.38–37.08)	41.40 (37.70–43.90)	**0.000**
Globin (g/L)	30.80 (27.73–32.55)	28.80 (26.00–32.50)	0.227
Total protein (g/L)	65.25 (61.13–69.38)	70.10 (66.60–73.7)	**0.000**
LDH (U/L)	192.00 (168.50–247.50)	183.00 (155.00–245.00)	0.304
ALP (U/L)	107.50 (75.50–122.25)	53.00 (43.00–67.00)	**0.000**
Urea (mmol/L)	2.88 (2.18–4.00)	3.40 (2.70–4.20)	**0.016**
Creatinine (μmol/L)	48.00 (42.25–53.75)	56.00 (50.00–60.00)	**0.000**
Coagulation profile	*n* = 20	*n* = 123	
PT (s)	13.10 (12.73–13.48)	13.50 (13.10–13.90)	**0.017**
APTT (s)	38.50 (33.20–40.05)	39.30 (37.30–41.58)	0.077
D-dimer (μg/mL FEU)	1.31 (0.72–1.71)	0.27 (0.22–0.51)	**0.000**
Cytokines	*n* = 16	*n* = 133	
IL-6 (pg/mL)	6.24 (2.05–38.78)	1.74 (1.50–5.37)	**0.004**
IL-8 (pg/mL)	8.35 (5.08–13.93)	7.40 (5.00–12.10)	0.608
TNF-α (pg/mL)	6.85 (5.00–8.30)	6.40 (5.25–7.65)	0.963
IL-1b (pg/mL)	5.00 (5.00–6.20)	5.00 (5.00–5.00)	0.381
IL-2R (U/mL)	332.50 (250.75–389.00)	317.00 (254.00–461.00)	0.755

*WBC* white blood cell, *hs-CRP* high-sensitivity C-reactive protein, *ALT* alanine aminotransferase, *TBIL* total bilirubin, *DBIL* direct bilirubin, *IBIL* indirect bilirubin, *AST* aspartate aminotransferase, *LDH* lactic dehydrogenase, *ALP* alkaline phosphatase, *PT* prothrombin time, *APTT* activated partial thromboplastin time, *IL* interleukin, *TNF* tumor necrosis factor

^a^The median for continuous variables in two independent groups were tested by Wilcoxon rank-sum test for data with skewed distribution. Bold figures indicate *p* values < 0.05

Among the 20 pregnant patients in our cohort, two terminated their pregnancy for personal reasons and another two were lost to follow-up. The obstetric and perinatal outcomes of the remaining 16 cases are listed in Table 2. Only one neonate was delivered before 37 weeks, and the rest all reached full term. With the concern of vertical transmission, 82% (27) of the neonates were delivered by Cesarean section. One neonate was tested SARS-CoV-2 polymerase chain reaction (PCR) positive at 36 h after delivery; however, umbilical cord blood and placenta both tested SARS-CoV-2 negative.^42^ The newborn was transferred to Wuhan Children’s Hospital after a confirmed diagnosis. The SARS-CoV-2 test turned negative at 15 days after birth, and the baby was discharged the day after when the chest computed tomography showed improvement of viral pneumonia.

**Table 2:** Obstetric and perinatal outcomes

	COVID-19 (*n* = 17)	Control (*n* = 121)^a^	*p*^b^
Gestational age on delivery, mean ± SD (weeks^+days^)	39 ± 9.9	39 ± 0.7	0.111
Mode of delivery, *n* (%)			**0.000**
Cesarean delivery	16 (100)	57 (47)	
Vaginal delivery	0 (0)	64 (53)	
Preterm delivery, *n* (%)	1 (5)	7 (6)	0.640
Full-term delivery, *n* (%)	15 (95)	114 (94)	0.640
Clinical outcomes of neonates	*n* = 16	*N* = 121	
Neonatal birth weight (mean ± SD), g	3240.3 ± 391.5	3317.1 ± 455.3	0.509
Confirmed neonatal SARS-CoV-2, *n* (%)	1 (6)		
Transferred to NICU, *n* (%)	4 (25)		
Perinatal death, *n* (%)	0 (0)		
Stillbirth, *n* (%)	0 (0)		
Neonatal death, *n* (%)	0 (0)		

*SD* standard deviation

^a^Obstetric and perinatal outcomes of healthy pregnant controls were collected from published data in Wuhan in 2020^73^. ^b^The mean for continuous variables in two independent groups were tested by t test. Bold figures indicate *p* values < 0.05

### Immune cell composition differs in pregnant COVID-19 patients

**Fig. 1:**
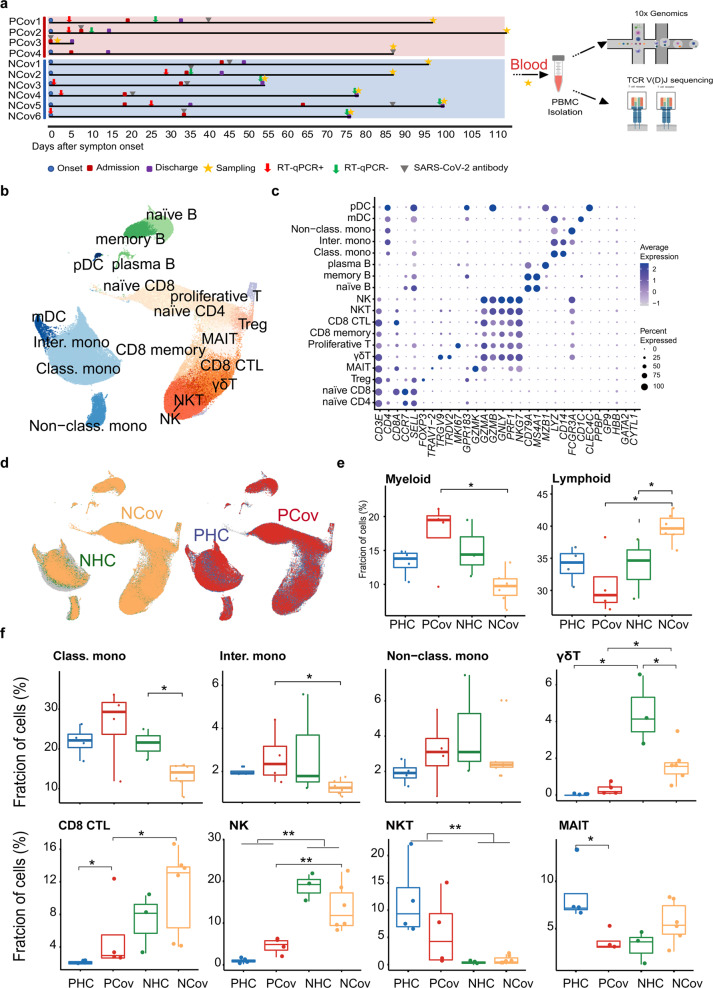
Single-cell atlas and cell composition of recovered COVID-19 patients and healthy controls. **a** Sample collection time for four pregnant patients and six non-pregnant patients recovered from SARA-CoV-2 infection and the overall study design. Onset indicates the onset of COVID-19-related symptoms; RT-qPCR+ indicates days when subject’s PCR tested positive for SARS-CoV-2 in nasopharyngeal or sputum samples, while RT-qPCR− indicates the days when subjects tested negative for infection. Sampling indicates the days when the blood samples were collected for single-cell sequencing. SARS-CoV-2 antibody indicates the days when the donors tested positive for SARS-CoV-2-specific IgM and IgG. **b** Integrated UMAP graph of 172,988 cells derived from our study, color-coded by cell types. **c** Dot plot showing expression level of canonical cell markers used to assign cell identities. The color scale indicates the expression levels and the size of dots is proportional to the fraction of cells. **d** UMAP projection of the pregnant or non-pregnant donors. PHC pregnant healthy controls (blue), PCov pregnant COVID-19 patients (red), NHC non-pregnant healthy controls (green), NCov non-pregnant COVID-19 patients (orange). **e** Percentages of myeloid cells and lymphoid in PHC (*n* = 4), PCov (*n* = 4), NHC (*n* = 3), and NCov (*n* = 6), color-coded by groups. **f** Fraction of eight cell types in the sequenced cells. Box plots show median, interquartile range (IQR), and the whiskers corresponding to the highest and lowest points within 1.5 times of IQR. Each dot represents an individual. *p < 0.05, **p < 0.01. Wilcoxon rank-sum test, adjusted for Bonferroni post hoc

To explore the differential immune responses in pregnant COVID-19 patients, we enrolled four pregnant patients (PCov) and six non-pregnant patients (NCov) to delineate pregnancy-associated immune responses against SARS-CoV-2. Blood samples were taken between 56 and 119 days after the symptom onset (Fig. 1a), at which period all subjects resolved their infection. Four pregnant healthy controls (PHC) with comparable gestational weeks (third trimester) and three non-pregnant female healthy controls (NHC) were enrolled in this study as control groups. The detailed demographics and clinical characteristics of these 17 individuals are shown in Supplementary Table 2, and the data of blood routine and laboratory tests collected at admission and sample collection time are listed in Supplementary Table 3. We performed single-cell mRNA sequencing and TCR sequencing of peripheral blood mononuclear cells (PBMCs) for all these subjects (Fig. 1a). In total, we obtained single-cell mRNA-seq data of 97,527 cells from 10 COVID-19 patients and 68,682 cells from the healthy controls.

Using Uniform Manifold Approximation and Projection (UMAP), 17 clusters of immune cells were identified according to the canonical marker genes (Fig. 1b, c and Supplementary Fig. 2a, b). Innate immune cells were classified into classical monocyte (*LYZ*, *CD14*^hi^, *FCGR3A*^−^), non-classical monocyte (*LYZ*, *FCGR3A*^hi^), intermediate monocyte (*CD14*^hi^, *FCGR3A*^+^), NK cell (*GNLY*, *NKG7*), myeloid DC (*CD1C*), and pDC (*CLEC4C*) dendritic cells. T cells (*CD3E*) were classified into nine classes, including naive CD4 T cell (*CD4*), naive CD8 T cell (*CD8A*, *CCR7*), CD8 cytotoxic lymphocyte (CD8 CTL; *CD8A*, *GZMA*), Treg (*SELL*), effector memory CD8 T cell (CD8 TEM, *GZMB*, *PRF1*), MAIT (*TRAV1-2*), γδ T cell (*TRGV9*, *TRDV2*), proliferative T lymphocyte (*MKI67*), and NKT cell (*NKG7*). B cells (*CD79A*) were classified into naive B cell (*MS4A1*, *CCR7*, *SELL*), memory B cell (*MS4A1*), and plasma cell (*MZB1*).

We quantified cell composition among the PCov, NCov, PHC, and NHC (Fig. 1d–f and Supplementary Fig. 2c). Higher percentages of myeloid cells were observed in PCov compared to that in NCov, but no difference was found in the healthy controls. In contrast, fewer lymphoid cells were found in PCov (Fig. 1e), indicating that the sign of lymphopenia remained till the recovery phase. The proportion of intermediate monocyte was higher in PCov than in NCov (*p* = 0.038, Wilcoxon rank-sum test, below the same) and the trend was also found in classical monocyte (Fig. 1f). γδ T cells were markedly reduced in NCov compared to that in NHC (*p* = 0.045); in contrast, similarly low levels were found in both the PCov and PHC groups. Compared to NCov, PCov showed decreased CD8 CTLs (*p* = 0.038). This trend was also observed when comparing PHC with NHC (*p* = 0.029). A higher level of NK cells was found in the non-pregnant individuals compared to the pregnant groups (NHC&NCov vs PHC&PCov, *p* = 0.000), while conversely a higher level of NKT cells was found in the pregnant groups (*p* = 0.004). Interestingly, the innate-like MAIT cells were found significantly reduced in PCov compared to PHC (*p* = 0.029); however, a reverse trend was seen in NCov.

### Pregnancy impact on the immune cell transcriptome in the COVID-19 recovery phase

**Fig. 2:**
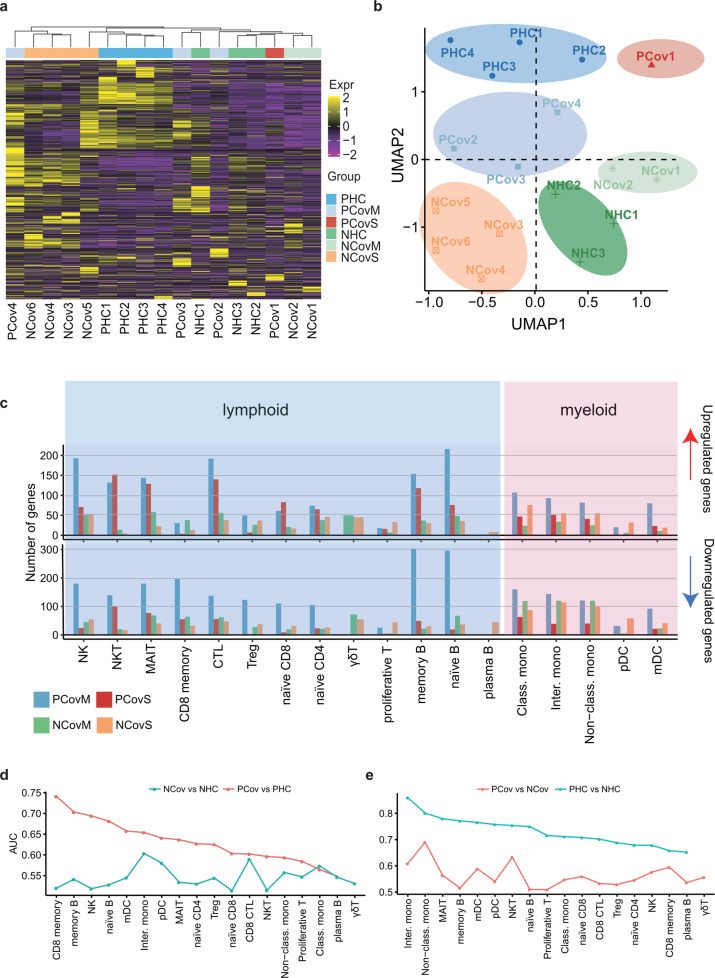
Pregnancy-associated immunity impact on anti-SARS-CoV-2 responses. **a** Heatmap of differentially expressed genes identified from pseudo-bulk gene analysis. The color scale indicates *Z* score-transformed expression level. **b** The UMAP projection of 17 donors based on the pseudo-bulk gene expression matrix presented in **a**. Samples were colored and shaded according to their assigned groups. Blue, pregnant patients who developed mild symptoms (PCovM); red, pregnant patient who developed severe symptoms (PCovS); green, non-pregnant patients who developed mild symptoms (NCovM); orange, non-pregnant patients who developed severe symptoms or had prolonged viral shedding (NCovS). **c** Upregulated or downregulated differentially expressed genes (DEGs) in each cell type of recovered COVID-19 patients compared to their corresponding healthy controls (PCovM and PCovS compared to PHC; NCovM and NCovS compared to NHC). Prioritizing the most affected cell types in response to COVID-19 (**d**) or pregnancy (**e**) by ranking the AUC scores derived from Augur algorithm^43^

To analyze how the transcriptome is affected by pregnancy in the COVID-19 recovery phase, we constructed a pseudo-bulk gene expression matrix for all samples, and only differentially expressed genes (DEGs) in the COVID-19 groups compared with their equivalent healthy controls were kept for further analysis. The hierarchical clustering of these DEGs showed that severe cases grouped together (Fig. 2a). The UMAP projection of these DEGs revealed six distinct subgroups, separated both by pregnancy status and severity (Fig. 2b). Specifically, the patients clustered into four subgroups, corresponding to their clinical manifestations (including severity, comorbidities, complications, and length of illness, Supplementary Table 2). PCov was divided into two subgroups, namely, PCovM (including moderate cases of PCov2/4 and asymptomatic PCov3) and PCovS (PCov1, severe case), while the NCov group were separated into NCovM (moderate cases NCov1/2) and NCovS [including severe NCov6, critical NCov4 cases, and 2 moderate cases of NCov3/4 with prolonged (>50 days) viral shedding time] (Supplementary Table 2). These results indicate that the severity of COVID-19 greatly impacts immune cell gene expression even in the recovery phase, irrespective of pregnancy status.

We therefore compared DEGs (*p* adjusted value cut-off of 0.05) between subgroups and their equivalent healthy controls (Fig. 2c). Myeloid cells generally had fewer DEGs than the lymphoid compartment, indicating that the myeloid compartment gradually returned to a steady state in the recovered phase. Among the lymphoid cells, fewer DEGs were identified in the plasma cells, proliferative T cell, γδ T, and pDC, whereas NK, NKT, CD8 CTL, and naive B and memory B cells had the most affected genes. There are notably more DEGs in multiple cell types of PCovM than all the other groups. NKT, MAIT, and CTL cells were highly more responsive in the pregnant patients than their non-pregnant counterparts. In addition, the number DEGs in memory B cells was also higher in the pregnant patients. In contrast, γδ T cells were more affected by COVID-19 in the non-pregnant patients.

To further prioritize the cell types most responsive to the SARS-CoV-2 infection or pregnancy, we applied Augur algothrim^43^ to our scRNA-seq data and found that CD8 memory cells and memory B cells had the highest scores comparing PCov and PHC (area under the curve (AUC) > 0.7), while intermediate monocyte and CD8 CTL may be affected the most in NCov compared to that in NHC (AUC > 0.6) (Fig. 2d). Comparing the pregnant and non-pregnant groups, we found that intermediate monocytes and non-classical monocytes had the highest scores (AUC > 0.8) between PHC and NHC, while non-classical monocytes, NKT, and intermediate monocytes likely differ the most in PCov compared to that in NCov (AUC > 0.6) (Fig. 2e).

### Elevated lymphoid response to COVID-19 despite marked lymphopenia in pregnancy

**Fig. 3:**
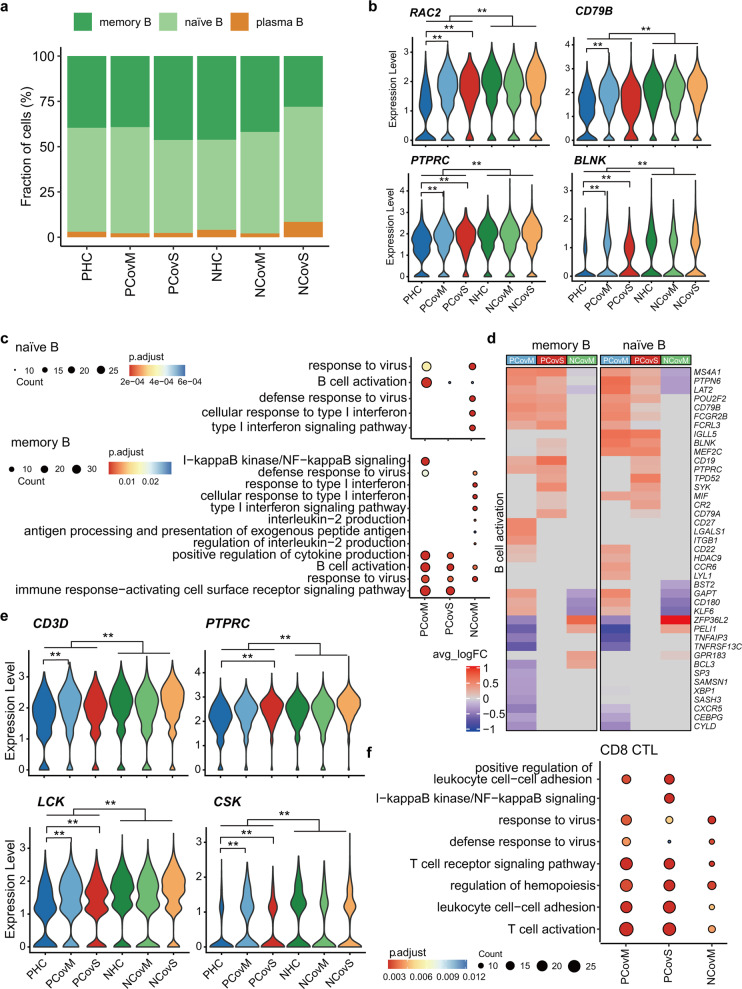
Enhanced activation in the lymphoid cells of pregnant COVID-19 patients. **a** Percentages of B cell subtypes from the PHC, PCovM, PCovS, NHC, NCovM, and NCovS groups. Violin plots showing the expression of genes involved in BCR signaling in memory B cell (**b**) or TCR signaling in CD8 CTL (**e**). *p < 0.05, **p < 0.01, Wilcoxon rank-sum test, adjusted for Bonferroni post hoc test. Top GO terms and pathways enriched for DEGs in naive B, memory B (**c**), or CD8 CTL (**f**) cells. Color scale indicates adjusted *p* values, which was derived from a hypergeometric test. The size of symbols is in proportion to gene counts enriched in the corresponding GO terms. **d** Heatmaps of DEGs enriched in “B cell activation” pathway for memory B and naive B cell cells. The color scale indicates the average log (fold change over the healthy controls) of representative genes

We first examined the lymphoid compartment in the recovered COVID-19 patients. The fractions within the B cell compartment were mostly similar among all groups, but a higher level of plasma cells was found in the NCovS group (Fig. 3a). Molecules involved in BCR signaling including *RAC2*, *CD79B*, *PTPRC*, and *BLNK* all showed lower expression in the pregnant groups but upregulated in PCov compared to PHC in both memory B (Fig. 3b) and naive B cells (Supplementary Fig. 3a). Correspondingly, DEGs between PHC and NHC was enriched in “B cell receptor signaling” pathway (Supplementary Fig. 3b). Moreover, in both memory and naive B cells, more DEGs were enriched in “B cell activation pathway” in pregnant group (PCov vs PHC) than non-pregnant group (NCov vs NHC) (Fig. 3c), with more upregulated genes (Fig. 3d). “Immune response-activating cell surface receptor signaling pathway” and “Positive regulation of cytokine production” were only enriched in memory B cells from the PCov groups (Supplementary Fig. 3c, d).

Similarly, molecules involved in TCR signaling, such as *CD3D*, *PTPRC*, *LCK*, and *CSK* had lower expression in CD8 CTLs of the pregnant groups (Fig. 3e). However, Gene Ontology (GO) analysis revealed that “T cell receptor signaling pathway,” “T cell activation,” and “leukocyte cell–cell adhesion” were also markedly more enriched in the PCov groups compared to that in PHC (Fig. 3f and Supplementary Fig. 3e, f). These results indicate that, despite a suppressed lymphoid response was established during pregnancy, the lymphoid compartment exhibited enhanced activation in response to SARS-CoV-2 infection.

### Normal TCR clonal expansion in response to COVID-19 in pregnancy

**Fig. 4:**
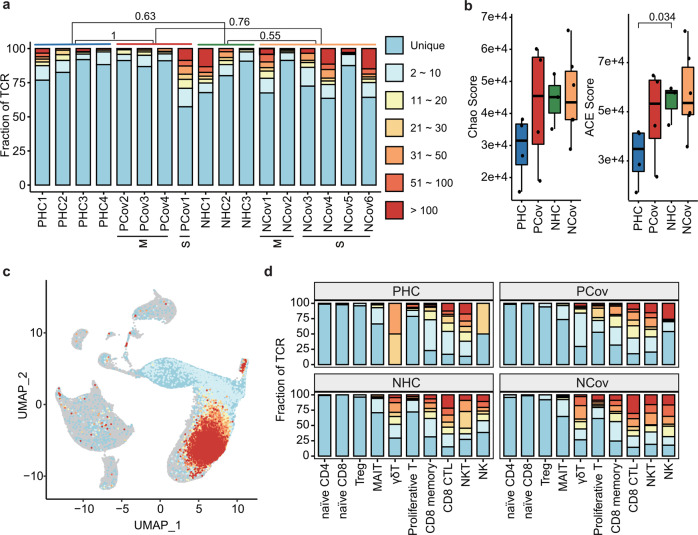
T cell clonality in response to COVID-19. **a** The frequency of TCR repertoires in all donors. The severity level is also shown. The frequencies of the expanded clones (clone ≥2) were compared among all the groups, Wilcoxon rank-sum test. **b** The clonal diversities differ between PHC and NHC while similar between PCov and NCov. The diversities were evaluated using the ACE and Chao indices (Borcherding et al.^71^). *p* Values of Wilcoxon rank-sum test are shown. PHC pregnant healthy controls (blue), PCov pregnant COVID-19 patients (red), NHC non-pregnant healthy controls (green), NCov non-pregnant COVID-19 patients (orange). **c** UMAP projection of TCR repertoires. **d** Percentages of TCR repertoires identified in the T cell subtypes

To further analyze difference in T cell response in pregnancy, different sizes of T cell clones were compared among the enrolled patients and healthy donors. The T cell clones showed comparable expansion between the recovered patients and the corresponding healthy controls (Fig. 4a). Albeit less T cell clone diversity was found in PHC compared with that in NHC, comparable diversities were found between PCov and NCov (Fig. 4b). We integrated the data of TCR sequencing and the scRNA-seq (Fig. 4c) and found CD8 CTL showed the highest proportions of large clones in all T cell subsets (Fig. 4d), in consistent with the previous studies.

The V(D)J gene rearrangements were analyzed to study the preferential gene usage of TCR. The top 10 complementary determining region 3 (CDR3) sequences of TRAV/TRAJ/TRBV/TRBJ showed a low level of similarity across all the groups (Supplementary Fig. 4a). We also identified high frequencies TCR combinations with TRBV27:TRBJ2-1 and TRBV20-1:TRBJ2-1 in PCov and TRAV29/DV5:TRAJ49 and TRBV9:TRBJ1-3 in NCov (Supplementary Fig. 4b). These recognition sequences may have distinct functions against SARS-CoV-2 infection.

### Higher tissue-homing profile in NK, NKT, and MAIT cells

**Fig. 5:**
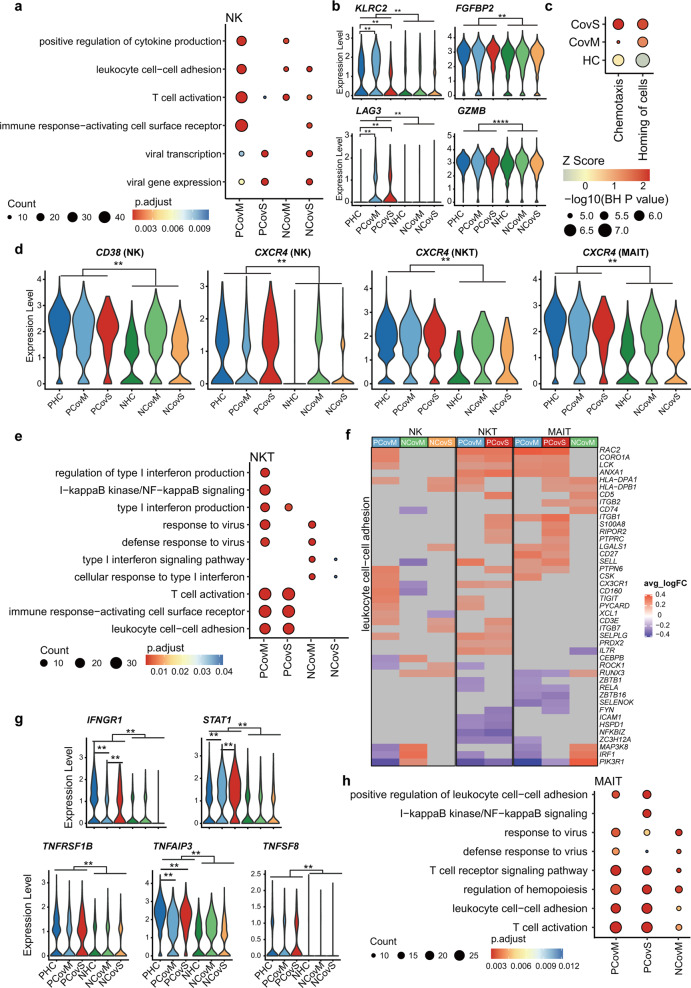
Pregnancy influences NK, NKT, and MAIT cells’ response in COVID-19. Top GO terms and pathways enriched for DEGs in NK (**a**), NKT (**e**), and MAIT (**h**) cells (PCovM and PCovS compared to PHC, NCovM and NCovS compared to NHC). Color scale indicates adjusted *p* values, which were derived from a hypergeometric test. The size of symbols is in proportion to gene counts enriched in the corresponding GO terms. **b** Violin plots showing the expression of representative markers for NK cells. **c** Representative pathways enriched for DEGs compared between pregnant and non-pregnant patients (PCovM vs NCovM and PCovS vs NCovS). Color scale indicates activation *Z* score provided by Ingenuity Pathway Analysis and the sizes of symbols were proportional to –log10 (*p* value adjusted by Benjamini and Hochberg correction). **d** Violin plots showing the expression of CD38 in NK cells and CXCR4 in NK, NKT, and MAIT by violin plot. **f** Heatmaps of DEGs enriched in “leukocyte cell–cell adhesion” pathway for NK, NKT, and MAIT cells. The color scale indicates the average log (fold change over healthy controls) of representative genes. **g** Violin plots showing representative gene expression in MAIT cells. **p < 0.01, ****p < 0.0001, Wilcoxon rank-sum test, adjusted for Bonferroni post hoc test

The fraction of NK cells was higher in the non-pregnant than in the pregnant groups (Fig. 1f); however, PCovM has more than three times more DEGs compared to PHC than NCov vs NHC (Fig. 2c), indicating that it is more responsive in the pregnant COVID-19 patients. Examining DEGs between the recovered patients and healthy control groups, we found that enrichment in “positive regulation of cytokine production,” “leukocyte cell–cell adhesion,” “T cell activation,” and “immune response-activating cell surface receptor” were more pronounced in PCovM (Fig. 5a and Supplementary Fig. 5a–c). KLRC2/NKG2C and FGFBP2/Ksp37, markers indicative of adaptive NK cells were higher in the pregnant groups, especially in PCovM, and the activation markers of *LAG3* and *GZMB* were also higher in the PCov groups (Fig. 5b). Looking into DEGs in NK cells between the PCov and NCov groups, we again found pathways of “chemotaxis” and “homing of cells” significantly activated in both PCovM and PCovS compared to their NCov counterparts (Fig. 5c). Molecules responsible for NK cell activation and chemotaxis, such as *CD38* and *CXCR4*, were higher in the pregnant compared to the non-pregnant patients, indicating that NK cells from PCov exhibit higher chemotaxis property (Fig. 5d).

Conversely, the pregnant groups generally have more NKT cells than the non-pregnant groups (Fig. 1f) and there are also more DEGs in NKT cells between PCov and PHC than NCov compared to NHC (Fig. 2b). GO terms of “immune response-activating cell surface receptor,” “T cell activation,” and “leukocyte cell–cell adhesion” were enriched in the PCov groups compared to PHC but not in NCov compared to NHC (Fig. 5e, f and Supplementary Fig. 5b, c). Similar to NK cells, *CXCR4* expression in NKT cells was markedly higher in the PCov groups (Fig. 5d).

MAIT cell is another group of innate-like T cells. It was found significantly reduced in PCov compared to PHC but not in NCov (Fig. 1f). MAIT cells in the pregnant groups show higher pro-inflammatory profile than the non-pregnant groups with higher expression level of *IFNGR1*, *STAT1*, and tumor necrosis factor (TNF)-related molecules, such as *TNFRSF1B*, *TNFAIP3*, and *TNFSF8* (Fig. 5g). Correspondingly, GO analysis showed that multiple immune pathways were more enriched in the PCov groups than in the NCov groups (Fig. 5h and Supplementary Fig. 5a–c) with no enrichment found for NCovS. Similar to NK and NKT cells, “leukocyte cell–cell adhesion” was pronouncedly enriched in MAIT cells of PCov groups than in the NCov groups compared to their corresponding healthy controls (Fig. 5f, h).

Taken together, NK, NKT and MAIT cells were more activated in the pregnant patients with enhanced chemotaxis signature than the non-pregnant patients, with notably higher expression of *CXCR4* in all three cell types (Fig. 5d).

### A heightened monocyte response in pregnant COVID-19 patients

The monocyte compartment was predicted to respond differently in pregnancy by Augur, correspondingly we found that activation makers of *CD83* and *FCGR1A* were elevated in the pregnant compared to the non-pregnant groups (Supplementary Fig. 6a), indicating a heightened monocyte response in pregnancy. Moreover, an overall higher expression level was observed in HLA-II molecules, such as *HLA-DRA*, *HLA-DPB1*, and *HLA-DMA*. These molecules were downregulated in three monocyte subsets from COVID-19 patients (Supplementary Fig. 6b) compared to the healthy controls, in line with previous reports.

**Fig. 6:**
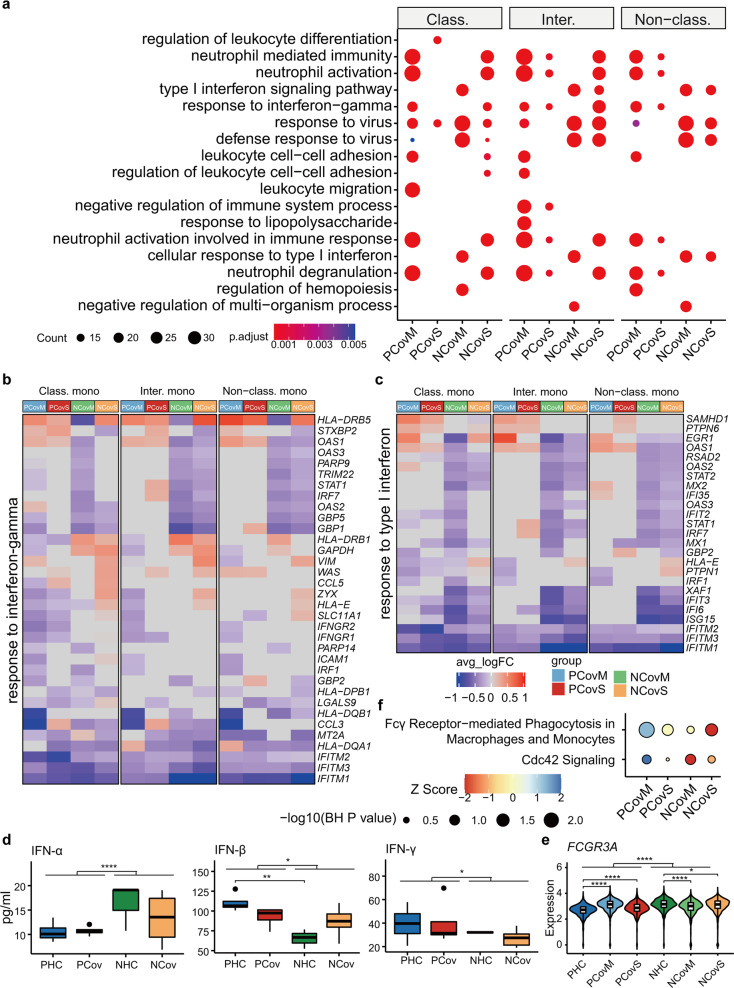
Pregnant COVID-19 patients have elevated monocytic response. **a** Top GO terms and pathways enriched for DEGs in classical, intermediate, and non-classical monocyte from recovered COVID-19 patients compared to respective healthy controls. The color scale indicates adjusted *p* values derived from a hypergeometric test. The size of symbols is in proportion to gene counts enriched in the corresponding GO terms. Heatmaps of DEGs enriched in “response to interferon-gamma” (**b**) and “response to type I interferon” (**c**). **d** Box plots showing the levels of IFN-α, IFN-β, and IFN-γ in the plasma of patients and healthy donors. Box plots show median, interquartile range (IQR), and the whiskers corresponding to the highest and lowest points within 1.5 times of IQR. Each dot represents an individual. *p < 0.05, **p < 0.01, ****p < 0.0001, one-way ANOVA test, adjusted for Bonferroni post hoc test. **e** Expression level of FCGR3A in non-classical monocytes. *p < 0.05, ****p < 0.0001, Wilcoxon rank-sum test, adjusted for Bonferroni post hoc test. **f** Phagocytosis-related pathways enriched for DEGs from non-classical monocytes. The color scale indicates activation *Z* score (compared to related healthy controls) provided by Ingenuity Pathway Analysis and the sizes of symbols were proportional to –log10 (*p* value adjusted by Benjamini and Hochberg correction)

We performed GO enrichment analyses on DEGs between the recovered COVID-19 patients and their respective healthy controls (Fig. 6a and Supplementary Fig. 6c). In addition, more genes involved in “type I interferon” and “interferon-gamma” pathway showed downregulation in COVID-19 patients (both PCov and NCov) (Fig. 6b, c). To understand whether this is related to the differences in plasma IFNs, we analyzed plasma IFN-α, IFN-β, and IFN-γ concentration from samples collected for scRNA-seq. Indeed, IFN-γ was higher in the pregnant groups compared to the non-pregnant groups (*p* = 0.017, one-way analysis of variance test), and PCov showed higher level of IFN-γ than NCov albeit not significant. The concentration of IFN-α and IFN-β, however, showed reverse trends (Fig. 6d). These results indicate a differential IFN response in pregnancy.

Interestingly, we also found that the expression level of *FCGR3A* in non-classical monocytes was elevated in PCov compared to that in PHC, whereas downregulated in NCov compared to that in NHC (Fig. 6e). Correspondingly, the phagocytosis-related pathways “CDC42 signaling” and “Fcγ receptor-mediated phagocytosis in macrophages and monocytes” was activated in the PCov groups while inhibited in the NCov groups (Fig. 6f), indicating that non-classical monocytes were more active in PCov than in NCov.

### Differential IFN responses in monocyte compartment during pregnancy

**Fig. 7:**
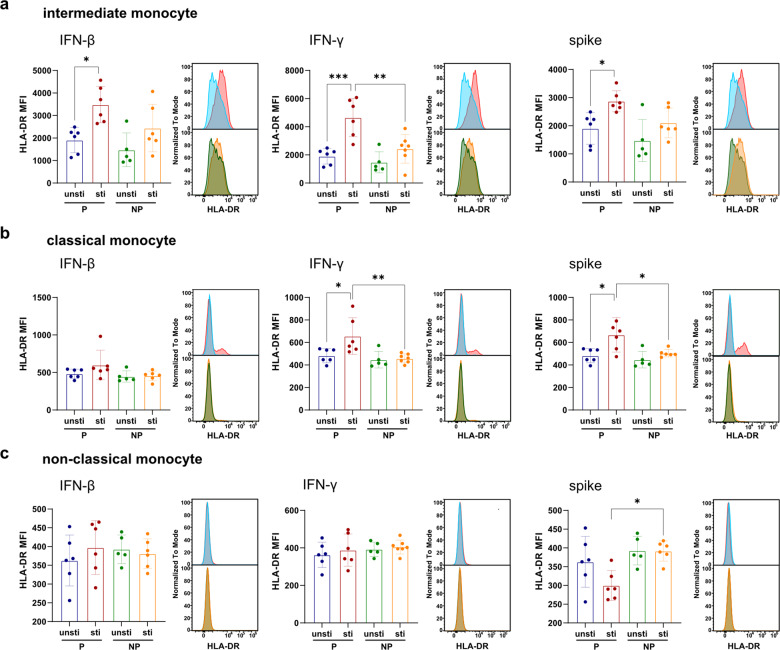
Validation of inflammatory features combined with IFN response of pregnant women by in vitro experiment. **a**–**c** Comparison of the MFI (mean fluorescence intensity) HLA-DR in intermediate, classical, and non-classical monocyte in stimulated compared to unstimulated blood samples by box plot and histogram. Freshly isolated blood samples withdrawn from pregnant (*n* = 6) and non-pregnant healthy controls (*n* = 6) were incubated with 1000 U/mL IFN-β, 1000 U/mL IFN-γ, or 25 ng/mL SARS-CoV-2 spike protein for 24 h. unsti unstimulated samples, sti stimulated samples. Barchart show median and interquartile range (IQR). Each dot represents an individual. Wilcoxon rank-sum test, adjusted for Bonferroni post hoc. *p < 0.05, **p < 0.01, ***p < 0.001

To further elucidate whether monocytes respond differently to type I and II IFNs in pregnancy, we performed in vitro stimulation assay by incubating the freshly isolated whole blood with IFN-β and IFN-γ for 24 h and analyzed monocyte response by flow cytometry. We found that intermediate monocytes from pregnant donors were more responsive to IFN-β and IFN-γ than non-pregnant female donors, evident by significant upregulation of HLA-DR in stimulated compared to unstimulated pregnant blood samples (Fig. 7a). Classical monocytes from pregnant donors were also more responsive to IFN-γ compared to the non-pregnant group but not IFN-β (Fig. 7b). Non-classical monocytes from both pregnant and non-pregnant donors did not upregulate HLA-DR expression upon IFN-β or IFN-γ treatment (Fig. 7c). We also analyzed CD83 and IFNGR1 expression and found that IFN-β and IFN-γ stimulation did not upregulate CD83 expression on all three monocyte subsets, whereas classical monocytes downregulated IFNGR1 level upon IFN-β and IFN-γ treatment (Supplementary Fig. 7).

To further investigate whether monocytes from the pregnant patients also respond differently to SARS-CoV-2 antigen, we used SARS-CoV-2 spike protein to stimulate blood samples collected from pregnant and non-pregnant donors. Both classical and intermediate monocytes from pregnant donors were more responsive to SARS-CoV-2 spike protein by upregulating HLA-DR (Fig. 7a–c). In contrast, non-classical monocytes from non-pregnant donors were more responsive to SARS-CoV-2 spike protein by upregulating CD83 (Supplementary Fig. 7).

## Discussion

To investigate the unique immunological features of COVID-19 during pregnancy, we performed single-cell mRNA sequencing and single-cell TCR sequencing for PBMCs isolated from the recovered pregnant and non-pregnant COVID-19 patients as well as their equivalent healthy controls. Our data present a unique feature in both innate and adaptive immune responses in pregnant patients recovered from COVID-19.

Similar to other respiratory viral infections, T cells and B cells play a prominent role in SARS-CoV-2 infection.^44^ The published data reported that patients with severe COVID-19 can have either insufficient or excessive T cell responses,^5,45^ indicating a protective role of proper T cell response in defense against SARS-CoV-2. Meanwhile, over 30% COVID-19 patients showed increased plasmablasts and proliferation of memory B cells.^4^ We observed prolonged lymphopenia in the pregnant patients, indicating potentially suppressed lymphoid response, and consistently, molecules involved in BCR or TCR signaling are generally downregulated in the pregnant groups. However, we also found that pathways related to T or B cell activation, migration, and virus defense were upregulated in the pregnant patients compared to the healthy control, indicating an adequate lymphoid response mount in the pregnant patients in defense against SARS-CoV-2 infection.

The healthy pregnant donors enrolled in our study showed less T cell repertoire than the non-pregnant healthy controls, and this is in line with previous studies showing less repertoire diversity in the third trimester.^46,47^ However, similar level of the clonal expansion was found between the PCov and NCov groups, further supporting our previous observation that the T cell compartment was functional during pregnancy.

Many innate immune cells, including monocyte and NK cells, and innate-like cells, NKT and MAIT cells, were reported important in viral infections.^48–55^ In COVID-19, NK cells were reportedly deceased but activated in moderate and severe cases.^56,57^ NKT deduction in the peripheral blood was reported in severe COVID-19 patients. MAIT was also reported associated with COVID-19 severity.^31^ In our study, pregnant patients showed decreased proportion of NK cells compared to the non-pregnant patients, while a lower level of MAIT was found in PCov compared to that in PHC. Interestingly, a noticeable activation and chemotaxis signature was found in NK, NKT, and MAIT cells in the pregnant patients, similar to the findings in H1N1 infection during pregnancy.^26^ NK cell homing to the site of infection is important for pathogen clearance,^54^ and it was reported that enriched NK cells was found in bronchoalveolar lavage from COVID-19 patients,^58^ therefore the reduced numbers of NK cells in pregnant patients might reflect a potential redistribution of these cells to the infected sites, which may contribute to shorter virus shedding time in the pregnant patients. Similarly, MAIT, which was referred as antimicrobial T cells and acted as innate-like sensors and mediators of antiviral responses,^59^ also have strong tissue-homing property.^60^ It is reported that MAIT decreased and activated profoundly in COVID-19 patients but recovered to normal levels in convalescence.^31,61^ Interestingly, we found MAIT cells depleted from the circulation of pregnant patients (compared to healthy pregnant controls) but remained activated. Collectively, we speculated that the higher chemotaxis signature identified in NK cells, NKT, and MAIT cells may be important in controlling the SARS-CoV-2 infection in pregnancy.

Dysregulated monocyte response has been reported to be involved in the pathogenesis and fueled the cytokines storm in COVID-19 patients.^29,62^ Increased classical monocyte proportion and activation evidenced by higher expression of monocyte-derived cytokines was found in the severe COVID-19 cases,^63^ whereas intermediate monocyte was found decreased.^64^ In our study, notably more classical and intermediate monocytes were found in PCov than in PHC while a reverse trend was found in the non-pregnant groups. The relative upregulation of activation markers and HLA-II molecules was found in the pregnant groups, indicating an elevated monocytic response during pregnancy. Similarly, enhanced monocytic response has been reported to contribute to inflammation following influenza A virus infection in pregnancy.^25^ A differential IFN responses was found in the transcriptome of monocytes from the pregnant patients and this was confirmed in our in vitro stimulation assay showing that both classical and intermediate monocytes were more responsive to IFN-γ during pregnancy and intermediate monocytes were also more active upon IFN-β treatment. Using SARS-CoV-2 spike protein, we showed that classical and intermediate monocytes from pregnant donors may be more responsive to SARS-CoV-2 antigens.

Collectively, we profiled transcriptomic changes in immune cells of the recovered pregnant COVID-19 patients and validated key findings using in vitro stimulation assay. Pregnant patients displayed a suppressed but functional lymphoid immune response against the infection. a heightened activation and chemotaxis profile in the tissue-homing NK, NKT, and MAIT cells, and a differential type I and II IFN response. These features may account for the low severity and mortality in the pregnant COVID-19 patients despite sustained lymphopenia throughout the infection. Despite the limitations, our data provided valuable insights into the unique immune responses in the pregnant patients affected by COVID-19, which may help better understand the vulnerability of pregnant women to SARS-CoV-2 infection and provide useful information on vaccination in this poorly studied population.

### Limitation of the study

While our research may provide valuable information for further investigation into how pregnant patients respond to COVID-19, there are still some limitations in this study. First, our samples were collected from the recovered patients, which cannot delineate the immune response during the acute infection. Second, our sample size was relatively small because hospitalized pregnant patients accounted for only a small fraction of the infected population, and only one severe pregnant patient was found in our study. Last but not the least, pregnancy presented dynamic immune changes at different gestational ages, which might affect the immune response to COVID-19. However, the pregnant patients enrolled in our study mostly developed symptoms in the second trimester and samples were collected in the third trimester.

## Materials and methods

### Patients and sample collection

For a retrospective study, 20 pregnant women and 159 non-pregnant women of reproductive age ranging from 15 to 40 years with confirmed SARS-CoV-2 infection who were admitted to the Tongji Hospital between January 28 and May 9, 2020 were enrolled in our study. COVID-19 was confirmed according to the Seventh Chinese guideline. In Wuhan, quantitative real-time PCR testing for the diagnosis of SARS-CoV-2 infection was not available before 27 January 2020 and was not yet used for routine screening before mid-February 2020. Therefore, some of the cases were confirmed by serum test positive for SARS-CoV-2-specific antibodies. Demographics, medical history, comorbidities, complications, laboratory results (including SARS-CoV-2 RNA testing results), and the prognosis was abstracted from electronic medical records.

For single-cell analysis, patients and healthy donors were recruited in Tongji hospital (Supplementary Table 2). The study was approved by Tongji Hospital Ethics Committee (ID: TJ-IRB2020502). Written informed consent was obtained from all participants.

### Laboratory measurements

#### Real-time reverse transcription PCR assay for SARS-CoV-2

The presence of SARS-CoV-2 in nasopharyngeal or sputum samples was detected using a COVID-19 Nucleic Acid Detection Kit according to the manufacturer’s protocol (Shanghai Huirui Biotechnology Co., Ltd). Viral RNA was extracted from samples using the QIAamp RNA Viral Kit (Qiagen). Primers and probe targeting the SARS-CoV-2 envelope gene were used and the sequences of primers and probe are as follows: forward, 5’-TCAGAATGCCAATCTCCCCAAC-3’; reverse, 5’-AAAGGTCCACCCGATACATTGA-3’; and the probe 5’CY5-CTAGTTACACTAGCCATCCTTACTGC-3’BHQ1. The amplifications were performed on Droplet Digital PCR system (Bio-Rad), with the following condition: 50 °C for 15 min, 95 °C for 3 min, followed by 45 cycles of 95 °C for 15 s and 60 °C for 30 s.

#### Blood routine

Blood samples were collected in the vacuum tubes with EDTA as anti-coagulant (BD, Cat. No.367841) and analyzed by Sysmex xe-5000 (Japan) according to the manufacturer’s instructions.

#### Serum biochemical test

Blood samples were collected in the coagulation-promoting vacuum tubes (BD, Cat. No.367812). Serum biochemical examination was performed on an automated analyzer (Abbott.cl6000).

#### Coagulation profile

Blood samples were collected in the tubes with sodium citrate (BD, Cat. No.363095). Prothrombin time and activated partial thromboplastin time were detected by the hair color substrate method, and D-dimer was detected by enzyme-linked immunosorbent assay (ELISA; Stago, STA-R Max system, France).

#### Cytokines

Blood samples were collected in the coagulation-promoting vacuum tubes (BD, Cat. No. 367955). Concentration of IL-6 (Roche Diagnostics), IL-1b, IL-2R, IL-8, IL-10, and TNF-α (DiaSorin) was assessed in serum samples, according to the manufacturer’s instructions on a fully automated analyzer (Siemens Immulite 1000, DiaSorin Liaison or Roche Diagnostics Cobas e602) according to the manufacturers’ instructions.

#### SARS-CoV-2 immunoglobulin M (IgM) and IgG antibodies

The IgM and IgG antibodies against SARS-CoV-2 in serum specimens were detected by ELISA (YHLO Biotech Co. Ltd Shenzhen, China), according to the manufacturer’s instructions. The recombinant antigens contain nucleoprotein and spike protein of SARS-CoV-2. The antibody levels were expressed as arbitrary units per mL (AU/mL). The results ≥10 AU/mL are reactive (positive).

### Whole-blood stimulation assay

Blood samples were collected from healthy pregnant donors of similar gestational age (32–39 weeks) and age-matched (27–35 years old) healthy non-pregnant female donors into vacuum tubes containing heparin sodium (BD, Cat. No.367812). One milliliter of the collected whole blood was diluted 1:1 with RPMI-1640 culture medium and distributed in 5 mL culture tubes (BBI. Cat. No.F600535-0001). Samples were incubated at 37 °C, CO_2_ 5% in the presence of 1000 U/mL IFN-γ (Peprotech, Cat. No.300-02), 1000 U/mL IFN-β (Peprotech, Cat. No.300-02BC), 25 ng/mL SARS-Cov-2 spike protein (Atagenix, Cat. No.ATMP02492COV), or without stimulant.

Twenty-four hours after stimulation, the cells were pelleted and treated with red blood cell lysis buffer (BD, Cat. No.555899), washed, and incubated with Fc block (BioLegend, Cat. No.422302) followed by fluorochrome-conjugated antibodies at 37 °C for 30 min. The list of the used antibodies is provided in Supplementary Table 4. After washing away the unbound antibodies with fluorescence-activated cell sorting (FACS) buffer (phosphate-buffered saline with 3% bovine serum albumin), the cells were resuspended in FACS and analyzed on a Cytoflex LX flow cytometer (Beckman Coulter). Anti-Mouse Ig and κ/Negative Control Compensation Particles (BD, Cat. No. 552843) were used for compensation. Up to 2 × 10^6^ live cells were acquired per each sample. The acquired data were analyzed using the Flowjo software (version 10.7.0).

### Enzyme-linked immunosorbent assay

The following cytokines were measured by ELISA: IFN-α (Elabscience, Cat. No. H6125), IFN-γ (Elabscience, Cat. No. H0108c), and IFN-β (Elabscience, Cat. No. H0085c), according to the manufacturer’s instructions. Data were acquired on microplate readers (Thermo Fisher).

### Single-cell mRNA sequencing and analysis

Two mL of blood were collected in the vacuum tubes with EDTA as anti-coagulant (BD, Cat. No. 367841). PBMC was isolated by gradient density centrifugation (Ficoll Pague Plus, GE Health). Single cells were prepared in the Chromium Single Cell Gene Expression Solution using the Chromium Single Cell 5′ Gel Bead, Chip, and Library Kits v2 (10× Genomics) as per the manufacturer’s protocol. In all, 8000–10,000 total cells were loaded to each channel with an average recovery of 5758 cells. The cells were then partitioned into Gel Beads in Emulsion in the Chromium instrument, where cell lysis and barcoded reverse transcription of mRNA occurred, followed by amplification, shearing, and 5′ adapter and sample index attachment. Libraries were sequenced on MGISEQ-2000 at BGI, Beijing, China. On average, 200 Gb of raw data were generated for each sample.

For the 10× Genomics sequencing data alignment and quantification, the sequencing data were processed using the CellRanger software (v3.0.1) with default parameters and the GRCh38 v3.0.0 human reference genome (downloaded from https://support.10xgenomics.com/single-cell-gene-expression/software/pipelines/latest/advanced/references). Next, raw gene expression matrices generated per sample were combined in R (v3.6.0) and converted to a Seurat object using the Seurat R package (v3.2.1).^65^ From this, all samples with <500 cells were removed. Then only the cells with >500 unique molecular identifiers (UMIs) and genes with >1000 UMIs across all cells were kept for further analyses. And the doublets were identified using scrublet (v0.2.1),^66^ and a total of 13,944 doublets were removed.

#### Clustering and annotation

From the remaining 172,988 cells, gene expression matrices were normalized to total cellular read count using linear regression as implemented in Seurat’s ScaleData function. Variably expressed genes were selected as having a normalized expression between 0.125 and 3 and a quantile-normalized variance exceeding 0.5. To reduce the dimensionality of this dataset, the resulting 2000 highly variable genes were summarized by principal component analysis (PCA). The statistically significant PCs were used for Harmony (v1.0)^67^ to remove the batch effect, and the two-dimensional UMAP was calculated from the first 10 Harmony matrix using RunUMAP with default parameters. Cell clusters in the resulting two-dimensional representation were annotated to known cell types according to the canonical marker genes. To prioritize cell types, we used “calculate_auc” from Augur (v1.0.2)^43^ comparing recovered patients to their controls or comparing pregnant and non-pregnant patients.

#### Pseudo-bulk expression analysis

The pseudo-bulk expression matrix was calculated by summing all the cells from each sample. Then the DEGs from the COVID-19 groups compared to their equivalent healthy controls were identified using DESeq2 (v 1.26.0),^68^ and the genes with absolute log2 fold change > 1 and adjusted *p* value < 0.05 were taken for further hierarchical clustering. PCA from FactoMineR (v2.3)^69^ was performed on normalized pseudo-bulk expression matrix, and the first 6 PCs were used for further UMAP dimensionality reduction using umap (v0.2.7.0).^70^

#### Single-cell level DEG analysis

To identify DEGs for each group of 17 cell types, we compared cells from COVID-19 patients to cells from healthy control using the Seurat FindMarkers function with default parameters. DEGs were required to have an average log fold change 0.25 higher than the average expression in the other subclusters and adjusted *p* value < 0.05 and a detectable expression in >10% of all cells from that subcluster. In total, a list of 1028 DEGs genes was identified.

#### GO analysis

GO biological process and pathway enrichment analyses were performed using enrichGO and compareCluster from clusterProfiler (v3.12.0).^71^ And the results were visualized using the ggplot2 R package (v3.3.0).

### Single-cell TCR sequencing and analysis

Full-length TCR V(D)J segments were enriched from amplified cDNA from 5′ libraries via PCR amplification using a Chromium Single-Cell V(D)J Enrichment Kit according to the manufacturer’s protocol (10× Genomics). Demultiplexing, gene quantification, and TCR clonotype assignment were performed using Cell Ranger (v.3.0.2) vdj pipeline with GRCh38 human genome as reference. In brief, a TCR diversity metric, containing clonotype frequency and barcode information, was obtained. For the TCR, only cells with at least one productive TCR α-chain (TRA) and one productive TCR β-chain (TRB) were kept for further analysis. Each unique TRA(s)–TRB(s) pair was defined as a clonotype. If one clonotype was present in at least two cells, cells harboring this clonotype were considered to be clonal and the number of cells with such pairs indicated the degree of clonality of the clonotype. The repertoire diversities were evaluated using ACE and Chao indices.^72^ Using barcode information, T cells with prevalent TCR clonotypes were projected on the UMAP plot.

### Statistical analysis

All statistical analysis were performed using R (v3.6.0) and SPSS 26.0 (IBM). Continuous variables were described as the mean ± standard deviation when normally distributed or median (interquartile range) when not, and categorical variables were described as numbers (percentage). The means of medians for continuous variables in two independent groups were tested by the Wilcoxon rank-sum test. For multiple group comparison, the *p* value was adjusted by Bonferroni post hoc test. Proportions for categorical variables were tested by the Chi-square test. Fisher exact test was used when the sample sizes were small.

## Supplementary information

supplementary tables

Supplementary materials

agreement of change of authors

## Data Availability

All raw and processed data have been deposited in the BIG Data Center (Beijing Institute of Genomics, Chinese Academy of Sciences) under accession number HRA000700.
