# Supplementary material for: Differential immune responses in pregnant patients recovered from COVID-19
Source: Signal Transduct Target Ther. 2021 Jul 29;6:289. doi: 10.1038/s41392-021-00703-3 (PMC8320317; doi:10.1038/s41392-021-00703-3)
Supplement: Supplementary file 2 — Supplementary materials [file 41392_2021_703_MOESM2_ESM.pdf]

Supplementary Materials for

Differential immune responses in pregnant patients recovered from COVID-19

Ge Chen[1]†, Yiming Zhang[2]†, Yaoyao Zhang[2]†, Bin Yang[1], Mengge Cui[1], Qiuyue Liao[1], Hanxiao Chen[2], Hualin Bai[1], Dashing Shang[2], Jing Chen[1], ChaoYang Sun[1], Haiyi Liu[1], Fengyuan Liu[3], Bin Mao[2], Guoqiang Sun[4], Jihui Ai[1*], Lu Chen[2*], Jing-wen Lin[2*], Kezhen Li[1*]

Correspondence to: tjkeke@126.com

This PDF file includes:

Supplementary Fig 1. The kinetics of blood routine and biochemistry results of pregnant and non-pregnant COVID-19 patients during hospitalization (related to table 1).

Supplementary Fig. 2. Marker genes and cell composition in each cell clusters (related to Fig. 1).

Supplementary Fig. 3. Heatmaps of genes enriched in representative pathway in naïve B, memory B and CD8 CTL cells (related to Fig.3).

Supplementary Fig. 4. CDR3 and T usage of COVID-19 patients (related to Fig.4).

Supplementary Fig. 5. Heatmaps of genes enriched in representative pathways for NK, NKT and MAIT cells (related to Fig.5).

Supplementary Fig. 6. Gene expression features of monocyte subsets and Reactome enrichment (related to Fig. 6).

Supplementary Fig. 7. Gating strategy and expression of activation markers of monocytes in whole blood stimulation assay (related to Fig.7).

Supplementary Table 1. Clinical symptoms of COVID-19 pregnant patients and non-pregnant COVID-19 patients included in the clinical study.

Supplementary Table 2. Demographic and clinical information of COVID-19 patients and healthy donors enrolled in the single-cell study.

Supplementary Table 3. Laboratory tests of COVID-19 patients and healthy donors included in the single cell study at admission and sample collection.

Supplementary Table 4. Antibodies used by in the whole blood stimulation assay.

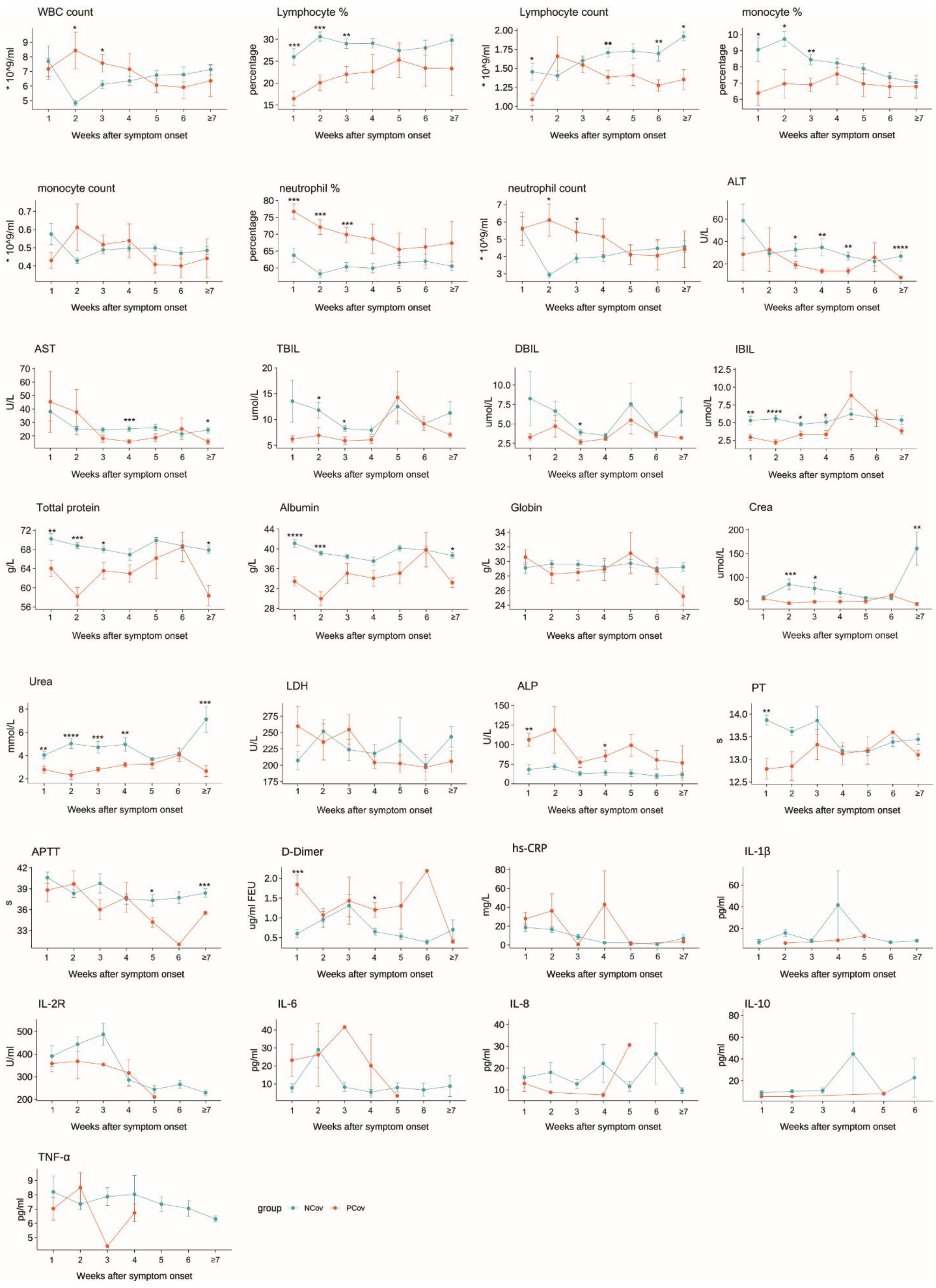
WBC count
Lymphocyte %
Lymphocyte count
monocyte %
monocyte count
neutrophil %
neutrophil count
ALT
AST
TBIL
DBIL
IBIL
Tottal protein
Albumin
Globin
Crea
Urea
LDH
ALP
PT
APTT
D-Dimer
hs-CRP
IL-1β
IL-2R
IL-6
IL-8
IL-10
TNF-α
Weeks after symptom onset
group NCov PCov

Supplementary Figure 1. The kinetics of blood routine and biochemistry results of pregnant and non-pregnant COVID-19 patients during hospitalization. Data presented as weeks after symptom onset (n>5). Multiple T test was used to determine significant difference between data collected from pregnant and non-pregnant female patients at the same time points. *p <0.05, **p <0.01, ***p<0.001, ****p <0.0001

WBC, White blood cell; hs-CRP, high sensitivity c-reactive protein; ALT, Alanine aminotransferase; TBIL, total bilirubin; DBIL, direct bilirubin; IBIL, indirect bilirubin; AST, Aspartate aminotransferase; LDH, Lactic dehydrogenase; ALP, Alkaline phosphatase; PT, Prothrombin time; APTT, Activated partial thromboplastin time; IL, interleukin; TNF, Tumor necrosis factor.

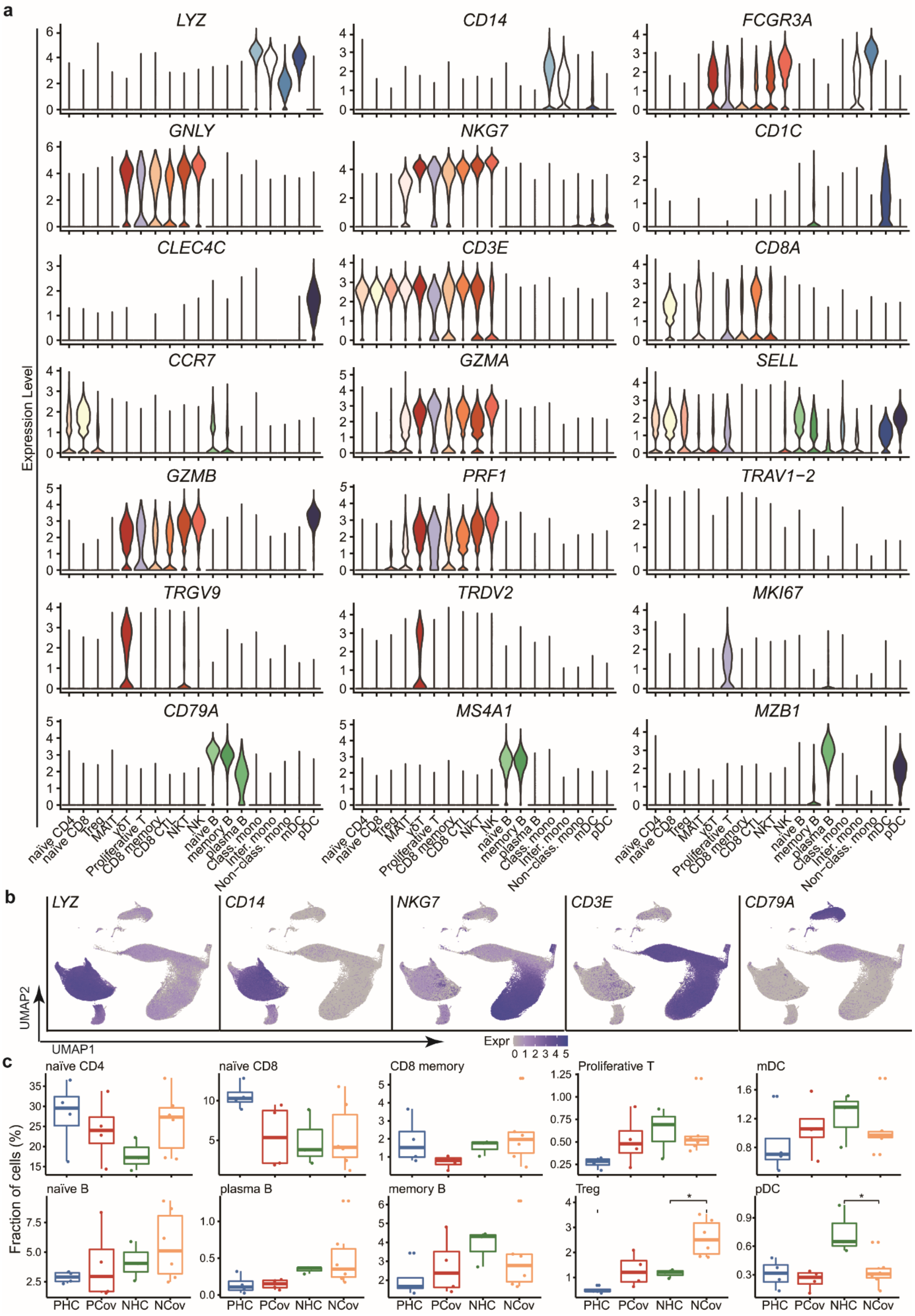

Supplementary Fig. 2. Marker genes and cell composition in each cell cluster (related to

Fig. 1).

(a) Violin plots showing expression of canonical marker genes in each cell type. (b) UMAP projection of the marker genes. The color scale indicates the expression level. (c). Proportion of cell types in pregnant healthy control (PHC, n = 4), PCov (pregnant COVID-19 patients, n = 4), NHC (non-pregnant healthy control, n = 3) and NCov (non-pregnant COVID-19 patients n = 6), color-coded by groups. Box plots show median, interquartile range (IQR) and the whiskers corresponding to the highest and lowest points within 1.5 times of IQR. Each dot represents an individual. *$p < 0.05$, **$p < 0.01$, Wilcox rank-sum test, adjusted for Bonferroni post hoc.

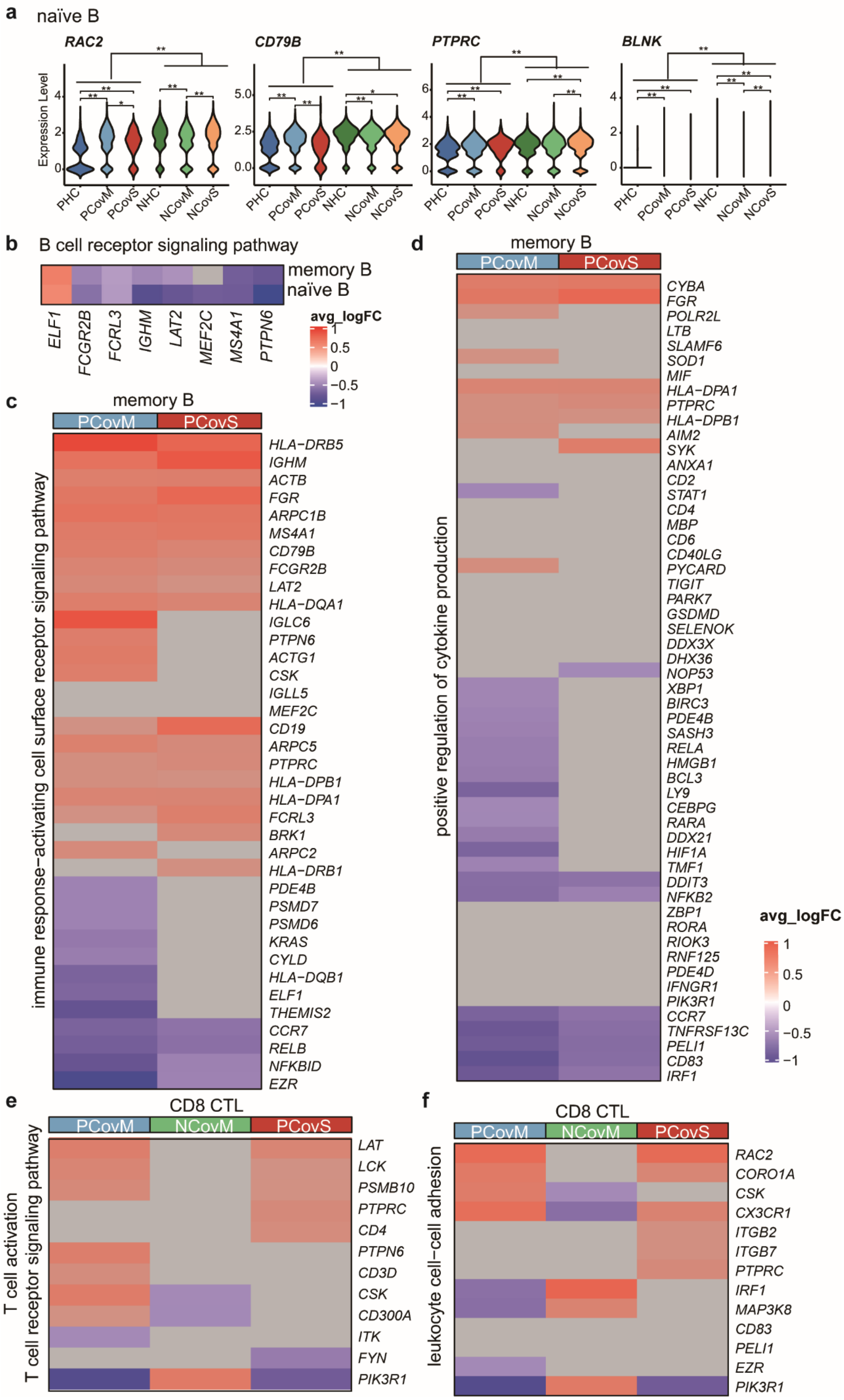
a
naïve B
RAC2
CD79B
PTPRC
BLNK
Expression Level
PHC
PCovM
PCovS
NHC
NCovM
NCovS
b
B cell receptor signaling pathway
memory B
naïve B
ELF1
FCGR2B
FCRL3
IGHM
LAT2
MEF2C
MS4A1
PTPN6
avg_logFC
c
memory B
PCovM
PCovS
immune response−activating cell surface receptor signaling pathway
HLA−DRB5
IGHM
ACTB
FGR
ARPC1B
MS4A1
CD79B
FCGR2B
LAT2
HLA−DQA1
IGLC6
PTPN6
ACTG1
CSK
IGLL5
MEF2C
CD19
ARPC5
PTPRC
HLA−DPB1
HLA−DPA1
FCRL3
BRK1
ARPC2
HLA−DRB1
PDE4B
PSMD7
PSMD6
KRAS
CYLD
HLA−DQB1
ELF1
THEMIS2
CCR7
RELB
NFKBID
EZR
d
memory B
PCovM
PCovS
positive regulation of cytokine production
CYBA
FGR
POLR2L
LTB
SLAMF6
SOD1
MIF
HLA−DPA1
PTPRC
HLA−DPB1
AIM2
SYK
ANXA1
CD2
STAT1
CD4
MBP
CD6
CD40LG
PYCARD
TIGIT
PARK7
GSDMD
SELENOK
DDX3X
DHX36
NOP53
XBP1
BIRC3
PDE4B
SASH3
RELA
HMGB1
BCL3
LY9
CEBPG
RARA
DDX21
HIF1A
TMF1
DDIT3
NFKB2
ZBP1
RORA
RIOK3
RNF125
PDE4D
IFNGR1
PIK3R1
CCR7
TNFRSF13C
PELI1
CD83
IRF1
avg_logFC
e
CD8 CTL
PCovM
NCovM
PCovS
T cell activation
T cell receptor signaling pathway
LAT
LCK
PSMB10
PTPRC
CD4
PTPN6
CD3D
CSK
CD300A
ITK
FYN
PIK3R1
f
CD8 CTL
PCovM
NCovM
PCovS
leukocyte cell−cell adhesion
RAC2
CORO1A
CSK
CX3CR1
ITGB2
ITGB7
PTPRC
IRF1
MAP3K8
CD83
PELI1
EZR
PIK3R1

Supplementary Fig. 3. Heatmaps of genes enriched in representative pathway in naïve B, memory B and CD8 CTL cells (related to Fig.5).

(a) Violin plots showing expression level of genes involved in BCR signaling in naïve B cell. (b) Heatmaps of DEGs between PHC and NHC enriched in 'B cell receptor signaling pathway' in memory B and naïve B cells. Heatmaps of DEGs enriched in 'immune response-activating cell surface receptor signaling pathway' (c) and 'positive regulation of cytokine production' (d) in memory B cells. Heatmaps of DEGs enriched in 'T cell activation' pathway (e), and 'leukocyte cell-cell adhesion' (f) in CD8 CTL cells. The color scale indicates average log (Fold Change over healthy controls) of representative genes. $^*p < 0.05$, $^{**}p < 0.01$. Wilcox rank-sum test, adjusting for the Bonferroni post hoc test.

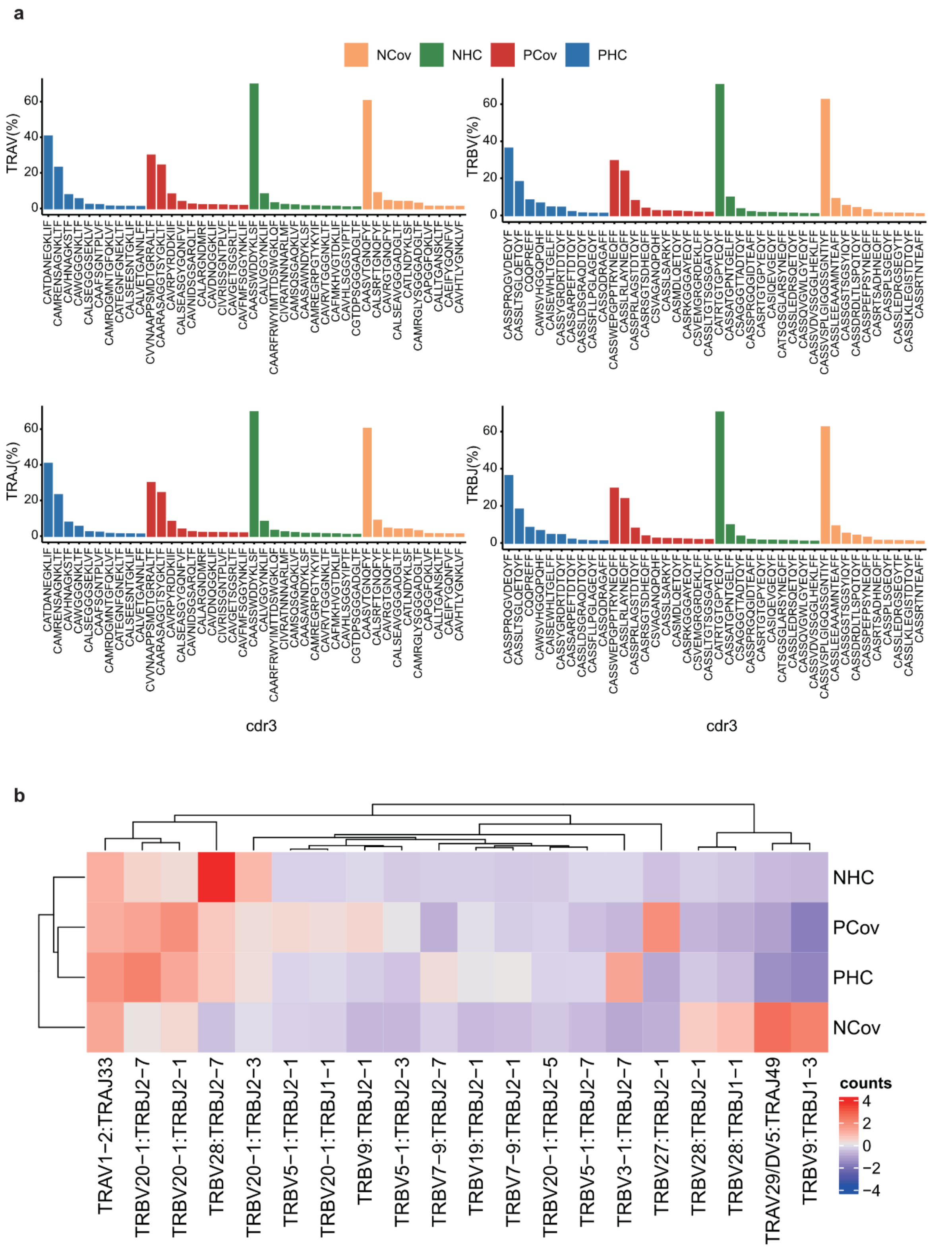

a
NCov
NHC
PCov
PHC
TRAV(%)
TRBV(%)
TRAJ(%)
TRBJ(%)
cdr3
cdr3
b
NHC
PCov
PHC
NCov
TRAV1−2:TRAJ33
TRBV20−1:TRBJ2−7
TRBV20−1:TRBJ2−1
TRBV28:TRBJ2−7
TRBV20−1:TRBJ2−3
TRBV5−1:TRBJ2−1
TRBV20−1:TRBJ1−1
TRBV9:TRBJ2−1
TRBV5−1:TRBJ2−3
TRBV7−9:TRBJ2−7
TRBV19:TRBJ2−1
TRBV7−9:TRBJ2−1
TRBV20−1:TRBJ2−5
TRBV5−1:TRBJ2−7
TRBV3−1:TRBJ2−7
TRBV27:TRBJ2−1
TRBV28:TRBJ2−1
TRBV28:TRBJ1−1
TRAV29/DV5:TRAJ49
TRBV9:TRBJ1−3
counts

Supplementary Fig. 4. CDR3 and TCR usage of COVID-19 patients

(a) The top 10 CDR3 usages are shown. (PHC, n = 4), PCov (pregnant COVID-19 patients, n = 4), NHC(n=3) and NCov (non-pregnant COVID-19 patients n = 6). (b) Differences of TRA/B rearrangement in different groups. The colors scale indicates the usage counts of specific V-J gene pairs.

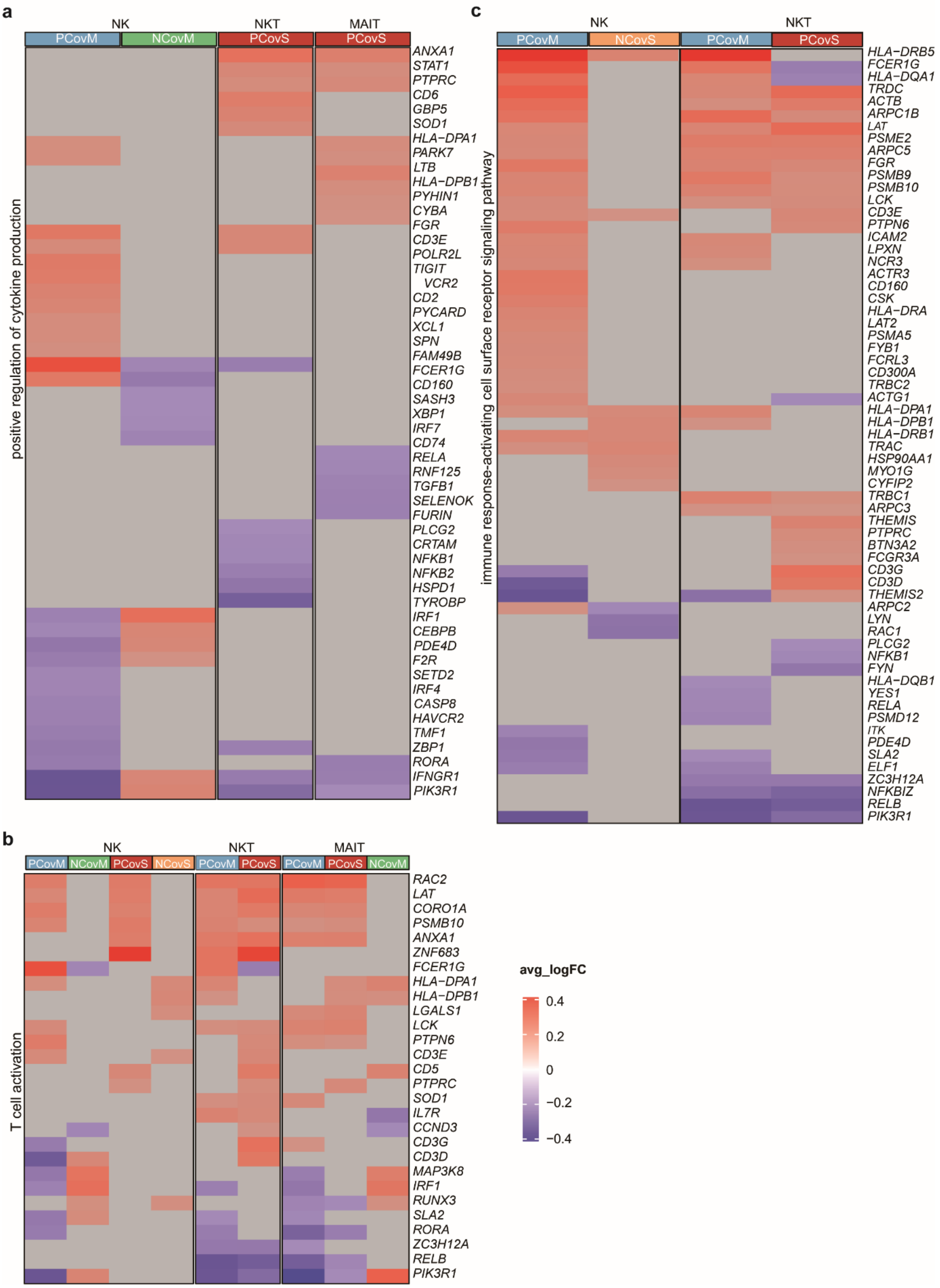
a
NK
NKT
MAIT
PCovM
NCovM
PCovS
PCovS
positive regulation of cytokine production
ANXA1
STAT1
PTPRC
CD6
GBP5
SOD1
HLA−DPA1
PARK7
LTB
HLA−DPB1
PYHIN1
CYBA
FGR
CD3E
POLR2L
TIGIT
VCR2
CD2
PYCARD
XCL1
SPN
FAM49B
FCER1G
CD160
SASH3
XBP1
IRF7
CD74
RELA
RNF125
TGFB1
SELENOK
FURIN
PLCG2
CRTAM
NFKB1
NFKB2
HSPD1
TYROBP
IRF1
CEBPB
PDE4D
F2R
SETD2
IRF4
CASP8
HAVCR2
TMF1
ZBP1
RORA
IFNGR1
PIK3R1
c
NK
NKT
PCovM
NCovS
PCovM
PCovS
immune response-activating cell surface receptor signaling pathway
HLA−DRB5
FCER1G
HLA−DQA1
TRDC
ACTB
ARPC1B
LAT
PSME2
ARPC5
FGR
PSMB9
PSMB10
LCK
CD3E
PTPN6
ICAM2
LPXN
NCR3
ACTR3
CD160
CSK
HLA−DRA
LAT2
PSMA5
FYB1
FCRL3
CD300A
TRBC2
ACTG1
HLA−DPA1
HLA−DPB1
HLA−DRB1
TRAC
HSP90AA1
MYO1G
CYFIP2
TRBC1
ARPC3
THEMIS
PTPRC
BTN3A2
FCGR3A
CD3G
CD3D
THEMIS2
ARPC2
LYN
RAC1
PLCG2
NFKB1
FYN
HLA−DQB1
YES1
RELA
PSMD12
ITK
PDE4D
SLA2
ELF1
ZC3H12A
NFKBIZ
RELB
PIK3R1
b
NK
NKT
MAIT
PCovM
NCovM
PCovS
NCovS
PCovM
PCovS
PCovM
PCovS
NCovM
T cell activation
RAC2
LAT
CORO1A
PSMB10
ANXA1
ZNF683
FCER1G
HLA−DPA1
HLA−DPB1
LGALS1
LCK
PTPN6
CD3E
CD5
PTPRC
SOD1
IL7R
CCND3
CD3G
CD3D
MAP3K8
IRF1
RUNX3
SLA2
RORA
ZC3H12A
RELB
PIK3R1
avg_logFC
0.4
0.2
0
−0.2
−0.4

Supplementary Fig. 5. Heatmaps of genes enriched in representative pathways for NK, NKT and MAIT cells (related to Fig. 5).

Heatmaps of DEGs enriched in ‘positive regulation of cytokine production’ (a), ‘immune response–activating cell surface receptor’ (b) and ‘T cell activation’ pathways (c) for NK, NKT and MAIT cells. The color scale indicates average log (Fold Change over healthy controls) of representative genes.

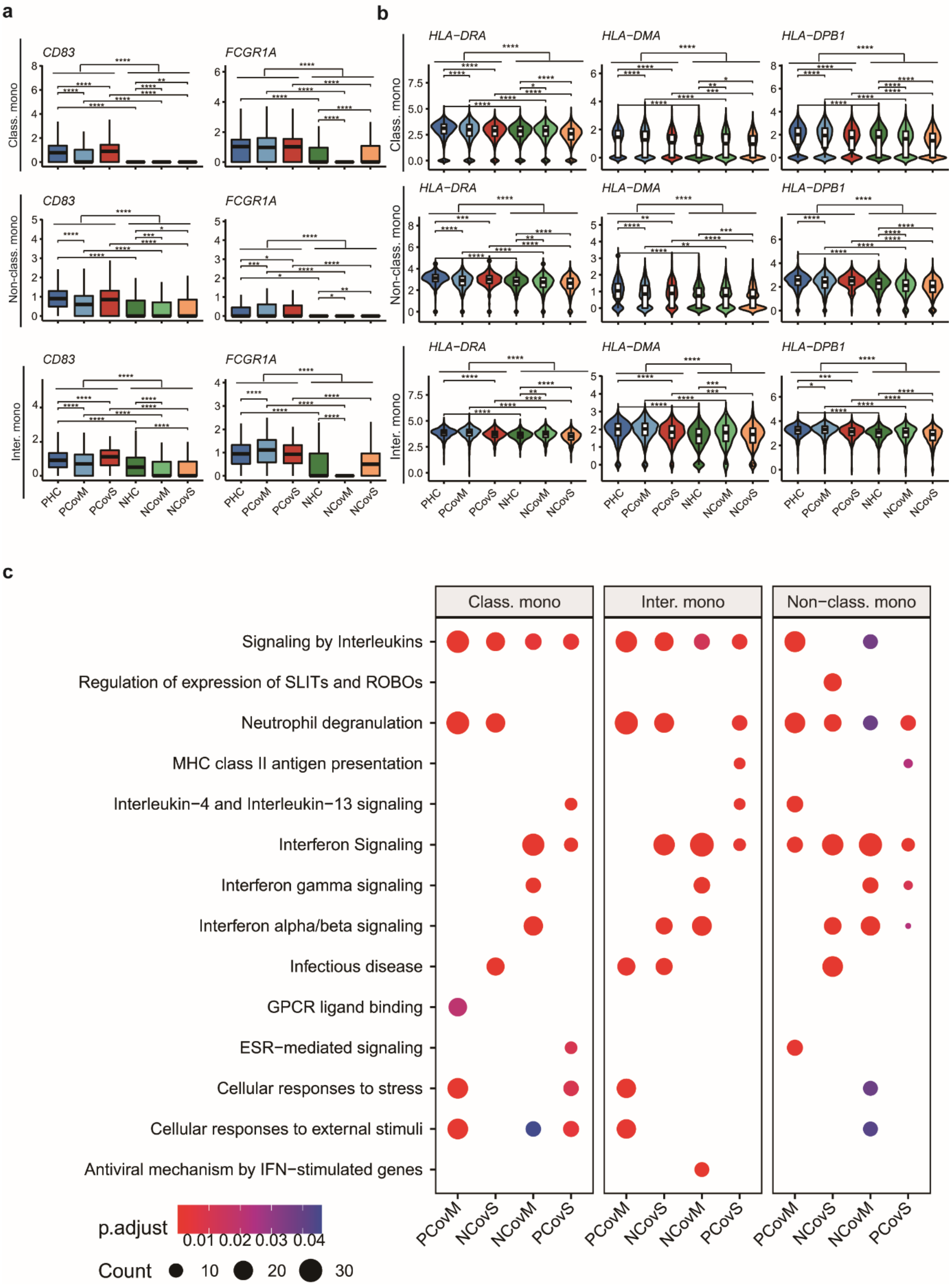
a
b
c
CD83
FCGR1A
HLA−DRA
HLA−DMA
HLA−DPB1
Class. mono
Non-class. mono
Inter. mono
PHC
PCovM
PCovS
NHC
NCovM
NCovS
Class. mono
Inter. mono
Non−class. mono
Signaling by Interleukins
Regulation of expression of SLITs and ROBOs
Neutrophil degranulation
MHC class II antigen presentation
Interleukin−4 and Interleukin−13 signaling
Interferon Signaling
Interferon gamma signaling
Interferon alpha/beta signaling
Infectious disease
GPCR ligand binding
ESR−mediated signaling
Cellular responses to stress
Cellular responses to external stimuli
Antiviral mechanism by IFN−stimulated genes
p.adjust
0.01 0.02 0.03 0.04
Count
10
20
30

Supplementary Fig. 6. Gene expression features of monocyte subsets and Reactome enrichment.

Box plots showing expression level of activation markers (a) and HLA-II molecules (b) in monocyte subsets. *$p < 0.05$, **$p < 0.01$, Wilcox rank-sum test. (c) Top Reactome terms and pathways enriched for DEGs in monocyte subsets (PCovM and PCovS compared to PHC, NCovM and NCovS compared to NHC). The color scale indicates adjusted P values derived from a hypergeometric test. The size of symbols is in proportion to gene counts enriched in the corresponding Reactome terms.

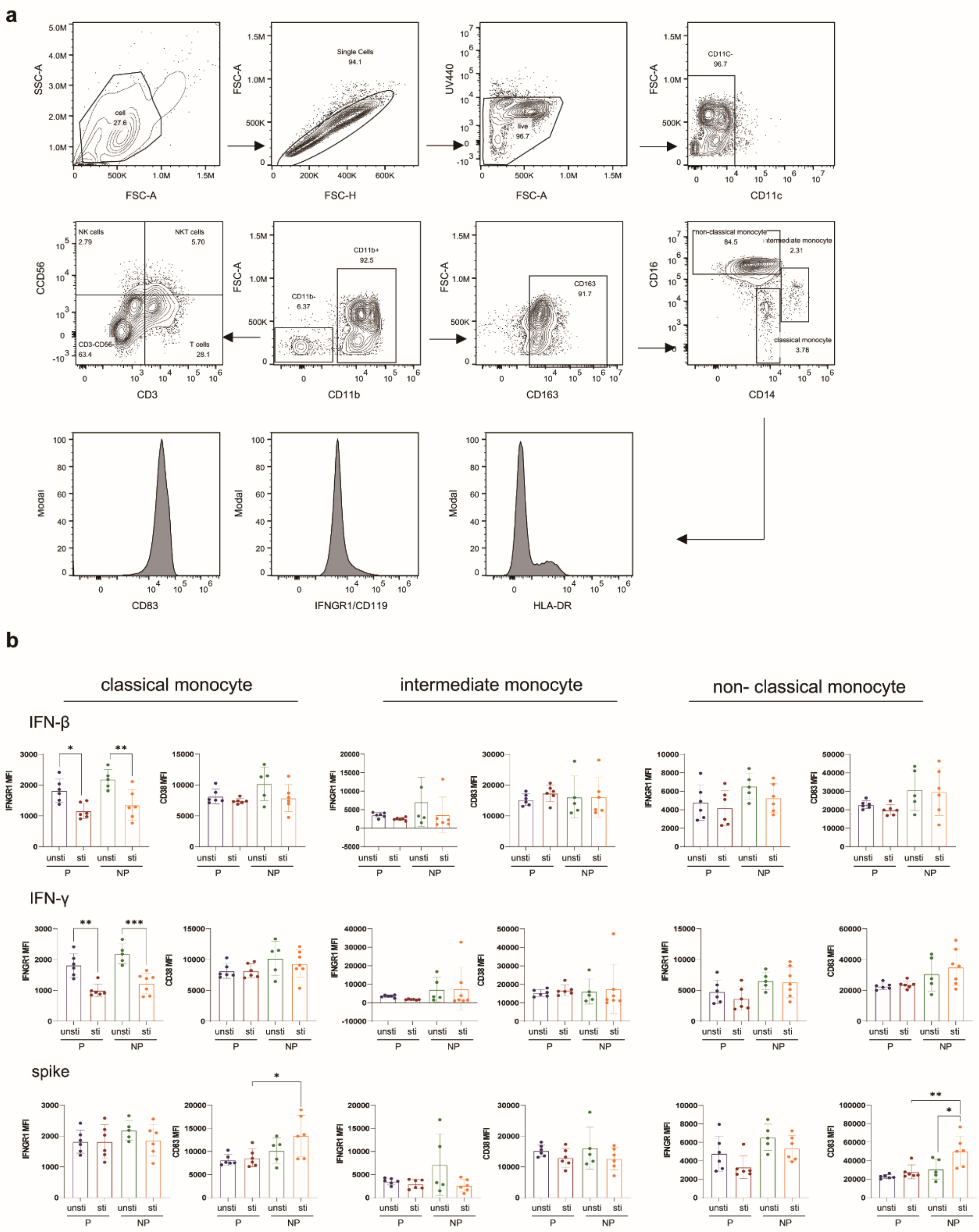

Supplementary Fig. 7. Gating strategy and expression of activation markers of monocytes in the whole blood stimulation assay (related to Fig. 7)

(a) Gating strategy used in the whole blood stimulation assay. (b) Comparison of the MFI (mean fluorescence intensity) of IFNGR1 and CD83 in intermediate, classical and non-

classical monocyte in stimulated compared to unstimulated blood samples. Freshly isolated blood samples withdrawn from pregnant (n=6) and non-pregnant healthy controls (n=6) were incubated with 1000U/ml IFN-β, 1000U/ml IFN-γ or 25ng/ml SARS-CoV-2 spike protein for or 24 hr. unsti, unstimulated samples; sti stimulated samples. Barchart show median, interquartile range (IQR). Each dot represents an individual. Wilcox rank-sum test, adjusted for Bonferroni post hoc. *p <0.05, **p <0.01, ***p <0.001.
