# Supplementary material for: Differential immune responses in pregnant patients recovered from COVID-19
Source: Signal Transduct Target Ther. 2021 Jul 29;6:289. doi: 10.1038/s41392-021-00703-3 (PMC8320317; doi:10.1038/s41392-021-00703-3)
Supplement: Supplementary file 3 — agreement of change of authors [file 41392_2021_703_MOESM3_ESM.pdf]

AGREEMENT OF CHANGE OF AUTHORS

After consultations, all the authors agreed with the change of author in this paper (title: Differential immune responses in pregnant patients recovered from COVID-19). We would like to change the author order as listed above.

Ge Chen[1]†, Yiming Zhang[2]†, Yaoyao Zhang[2]†, Bin Yang[1], Mengge Cui[1], Qiuyue Liao[1], Hanxiao Chen[2], Hualin Bai[1], Dashing Shang[2], Jing Chen[1], ChaoYang Sun[1], Haiyi Liu[1], Fengyuan Liu[3], Bin Mao[2], Guoqiang Sun[4], Jihui Ai[1*], Lu Chen[2*], Jing-wen Lin[2*], Kezhen Li[1*]

[11] Tongji Hospital, Tongji Medical College, Huazhong University of Science and Technology, Wuhan, Hubei, China

[2] Key Laboratory of Birth Defects and Related Diseases of Women and Children of MOE, State Key Laboratory of Biotherapy, West China Second University Hospital, Sichuan University and Collaborative Innovation Center for Biotherapy, Chengdu, Sichuan, 610041, China

[3] Biological Sciences Greenhouse, College of Art & Sciences, The Ohio State University, Columbus, OH, USA

[4] Department of Obstetrics, Hubei Maternal and Child Health Hospital, Wuhan, China

† These authors contributed equally to this study

Correspondence to: tjkeke@126.com (K.L.), lin.jingwen@scu.edu.cn (J-w.L.), luchen@scu.edu.cn

(L.C.), or jihuiai@tjh.tjmu.edu.cn (J.A.)

Signature:

Ge Chen, Hanxiao Chen, Guangqiang Sun

Bin Yang, Mengge Cui, Qinyue Liao

Hualan Bai, Jing Chen, Haiyi Liu

Jihui Ai, Fengyuan Liu, DaShing Shang

Yaoyao Zhang, Kezhen Li,

Zhang Yiming Jingwen Lin Chaoyang Sun

Bin Mao [illegible]

Date:

2021.5.31
